# Reproducibility of mass spectrometry based metabolomics data

**DOI:** 10.1186/s12859-021-04336-9

**Published:** 2021-09-07

**Authors:** Tusharkanti Ghosh, Daisy Philtron, Weiming Zhang, Katerina Kechris, Debashis Ghosh

**Affiliations:** 1 Colorado School of Public Health, University of Colorado, Anschutz Medical Campus, Aurora, USA; 2 Eberly College of Science, Penn State University, State College, USA; 3 Syneos Health, Morrisville, USA

**Keywords:** Reproducibility, Mass spectrometry, Metabolomics

## Abstract

**Background:**

Assessing the reproducibility of measurements is an important first step for improving the reliability of downstream analyses of high-throughput metabolomics experiments. We define a metabolite to be reproducible when it demonstrates consistency across replicate experiments. Similarly, metabolites which are not consistent across replicates can be labeled as irreproducible. In this work, we introduce and evaluate the use (Ma)ximum (R)ank (R)eproducibility (MaRR) to examine reproducibility in mass spectrometry-based metabolomics experiments. We examine reproducibility across technical or biological samples in three different mass spectrometry metabolomics (MS-Metabolomics) data sets.

**Results:**

We apply MaRR, a nonparametric approach that detects the change from reproducible to irreproducible signals using a maximal rank statistic. The advantage of using MaRR over model-based methods that it does not make parametric assumptions on the underlying distributions or dependence structures of reproducible metabolites. Using three MS Metabolomics data sets generated in the multi-center Genetic Epidemiology of Chronic Obstructive Pulmonary Disease (COPD) study, we applied the MaRR procedure after data processing to explore reproducibility across technical or biological samples. Under realistic settings of MS-Metabolomics data, the MaRR procedure effectively controls the False Discovery Rate (FDR) when there was a gradual reduction in correlation between replicate pairs for less highly ranked signals. Simulation studies also show that the MaRR procedure tends to have high power for detecting reproducible metabolites in most situations except for smaller values of proportion of reproducible metabolites. Bias (i.e., the difference between the estimated and the true value of reproducible signal proportions) values for simulations are also close to zero. The results reported from the real data show a higher level of reproducibility for technical replicates compared to biological replicates across all the three different datasets. In summary, we demonstrate that the MaRR procedure application can be adapted to various experimental designs, and that the nonparametric approach performs consistently well.

**Conclusions:**

This research was motivated by reproducibility, which has proven to be a major obstacle in the use of genomic findings to advance clinical practice. In this paper, we developed a data-driven approach to assess the reproducibility of MS-Metabolomics data sets. The methods described in this paper are implemented in the open-source R package *marr*, which is freely available from Bioconductor at http://bioconductor.org/packages/marr.

## Background

Metabolites are small molecules that represent a type of molecular phenotype that is intermediate between genetic and regulatory processes, such as methylation and transcription, and the physiological and disease state of an organism. Comprehensive profiling of the small molecule repertoire in a sample is referred to as metabolomics and has been applied extensively in detecting clinical biomarkers, studying physiological and disease processes, and predicting phenotypic changes [1, 2].

One of the most appealing features of metabolomics is the ability to characterize the full spectrum of metabolites by measuring them objectively and quantitatively. Metabolomics experiments can be classified into two categories: targeted and untargeted. Targeted metabolomics studies measure ions from biochemically known annotated metabolites. In contrast, untargeted metabolomics experiments measure the totality of ions in a set of predefined mass range [3, 4]. Among the platforms employed for measuring metabolites, Gas Chromatography Mass Spectrometry (GC–MS) and Liquid Chromatography Mass Spectrometry (LC–MS) are popular due to their sensitivity and coverage of all possible ions [5]. These GC–MS and LC–MS techniques prepare a sample at a high resolution, fragment it into ions and isolate the ions to generate spectra for the sample [6]. The fragmented ion spectra are subsequently reported on the basis of their physical properties (e.g., mass-charge ratio and retention time) [7, 8]. In many instances, some of the mass spectral signals measured by metabolomics experiments may not be biologically relevant due to background signals from the input sample preparation, or signals arising from the same analyte, such as isotopes and adducts [9]. Therefore, metabolite feature identification can sometimes be imperfect since noisy signals could be identified as a peak group [10]. Thus, many metabolomics data sets can have a large number of falsely identified metabolites or metabolite features with incorrect integration regions and missing values, which affect the reproducibility of the study [11, 12]. We use the term metabolites to refer to small compound features resulting from a metabolomics experiment in the rest of this article.

Reproducibility is an on-going challenge for high-throughput technologies developed in the last two decades for quantifying a wide range of biological processes. A persistent difficulty faced by researchers is the variability of output across replicate experiments [13]. Several authors have addressed the issue of reproducibility among high-throughput experiments [14–16]. In each high-throughput experiment (e.g., arrays, sequencing, mass spectrometry), a large number of features are measured simultaneously, and candidates are often subjected to follow-up statistical analysis. When measurements show consistency across replicate experiments, we define the signals that generate the measurements to be reproducible. Similarly, measurements that are not consistent across replicates may be problematic and those signals should be identified as irreproducible. In this work, metabolites that show consistency across MS-Metabolomics replicate experiments are termed reproducible and the ones that are not consistent are termed irreproducible. The reproducibility of a high-throughput experiment primarily depends on technical variables, such as run time, technical replicates (chemical factors and spike-in controls), laboratory operators, and biological variables, such as healthy and diseased subjects. A critical step toward making optimal design choices is to assess how these biological and technical variables affect reproducibility across replicate experiments [17, 18].

The simplest technique used to examine reproducibility is Spearman’s rank correlation across pairs of experiments, i.e., consistency of how metabolites are ranked in each experiment. However, the degree of reproducibility may depend on the magnitude of the signals, i.e., highly ranked signals are consistently more reproducible than low ranked signals. For this reason, Spearman’s rank correlation is not ideal for assessing the reproducibility of high-throughput experiments, since it does not account for the magnitude of signals.

The most detailed framework to assess reproducibility in high-throughput experiments was developed by [13]. Using Chromatin ImmunoPrecipitation sequencing (ChIP-seq) data as motivation, this procedure uses a copula mixture model in order to estimate the proportion of reproducible signals, and the Irreproducible Discovery Rates (IDR) for ChIP-seq peaks can be computed at each set of paired replicate ranks for assessing reproducibility and combining replicates. Several other methods have been proposed for characterizing and examining the reproducibility of high-throughput experiments, including the Correspondence at the Top (CAT) plot [19], which is a graphical tool to visualize and assess the reproducibility between a pair of replicate microarray experiments. However, CAT is only limited to visualization where the two ranked lists can be compared by looking at curves for the “proportion in common” based on fold change, between each experiment and a reference experiment. The choice of reference is important to calculate proportion in common. When the number of experiments is large, visualizing many curves with different reference experiments can be overwhelming. A more quantitative approach was a regression model developed to examine the effect of technical replicates for ChIP-seq and microarray data termed the correspondence curve regression [20]. The correspondence curve regression employs a cumulative regression model to quantify the simultaneous and independent effects of technical replicates on reproducibility without a specific significance threshold [21]. This regression model framework is a parametric alternative to existing graphical tools for comparing and benchmarking the reproducibility of different study designs in high-throughput sequencing experiments. Both IDR and correspondence curve regression are parametric approaches, and may be problematic if distributional assumptions are violated.

Like other high-throughput sequencing experiments (e.g., RNA-seq, ChIP-seq), reproducibility is also a major concern for effective downstream analysis of MS-Metabolomics data. There are few approaches that specifically examine reproducibility for MS-based metabolomics experiments. The most common approach is calculating the coefficient of variation (CV) across replicates. CV is computed by dividing the standard deviation of the replicates by the average, producing a measure for magnitude of the variation between replicates. An alternative measure RSD (Relative Standard Deviation) is often used instead of CV, where $\text {RSD}=100\times \frac{\hbox {Standard Deviation}}{\hbox {Absolute Mean}}$. It is often used to examine variation across biological and technical replicates samples and to filter metabolites [22]. The RSD (or CV) is often a poor predictor of feature quality because it only assesses variability across technical replicates, without considering biologically meaningful variability across subjects [23]. Typically, the RSD (or CV) is calculated across pooled QC samples for each feature and those with an RSD above a predetermined cutoff (e.g., 20–30%) are removed [24, 25]. That is, its properties are dependent on arbitrary cut-offs chosen by the user. In addition, RSD does not perform a statistical test for error control.

Limited research has been conducted comparing the reproducibility between biological or technical replicates. Even though previous research [15] has shown that the experiments were sufficiently reproducible between technical replicates, we strongly emphasize the need of further assessment of reproducibility of abundances of metabolites across both biological and technical replicates.

In this work, we present a method to identify reproducible metabolites for use with MS-Metabolomics data. We demonstrate that the (Ma)ximum (R)ank (R)eproducibility (MaRR) procedure can be adapted to high-throughput MS-Metabolomics experiments across (biological or technical) replicate samples. The MaRR procedure was originally developed by [26] to assess the reproducibility of RNA-seq data. It is a nonparametric approach that detects the change from reproducible to irreproducible signals. The advantage of using the MaRR procedure over model-based methods is that it does not make parametric assumptions on the underlying distributions or dependence structures of reproducible metabolites. Assessing reproducibility by the MaRR procedure will provide a new and robust way of studying reproducibility in the field of MS-Metabolomics experiments.

## Methods

### Maximum rank reproducibility (MaRR)

Maximum Rank Reproducibility (MaRR) was proposed to assess reproducibility of gene ranks in replicate experiments by [26]. Since, MaRR is a non-parametric procedure, it does not assume any distributional parameters on the underlying structure of reproducible features. The core idea behind developing MaRR is based on a maximum rank statistic, such that, the procedure has the ability to detect the transition from reproducible to irreproducible signals by minimizing the mean squared error between the observed and theoretical survival function. The survival function *S*(*t*), is the probability that a subject survives longer than time *t*. For this reason, the Survival function is often regarded as complementary cumulative distribution function.

Using the MaRR procedure, we propose to examine the reproducibility of ranked lists from replicate experiments and assess how concordant the metabolites are ranked in replicate experiments. Any numeric value for a metabolite feature, such as abundance, test statistic, *p*-value, *q*-value or fold change score can be used to rank the features. Then the method utilizes the ranks and not the original measurements. Additional file 1: Fig. S1 illustrates an example dataset of rank statistics for $M=10,000$ metabolites under the ideal setting of a perfect split (when the correlation between highly ranks signals is 1 and correlation between low ranked signals is 0).

Metabolites with reproducible measurements should be consistently highly ranked for both replicate experiments (blue, Additional file 1: Fig. S1), and are expected to have positive correlation in their ranks, whereas, metabolites with independent measures are assumed to have independent ranks and considered irreproducible (red, Additional file 1: Fig. S1).

### MS-metabolomics data design

We introduce notation to describe MS-Metabolomics data resulting from a study design with *D* layers $(D \ge 1)$. The layers in a study design may be technical (e.g., batches, technical replicates) or biological (e.g., disease status of subjects), and all layers measure abundances of metabolites. Under each layer in a MS-Metabolomics study design, multiple replicate experiments are obtained. Under each layer *d*, $d=1,2,\ldots ,D$, the reproducibility of these experiments are generally assessed across pairwise combinations of replicate experiments, i.e., ${n_d \atopwithdelims ()2}$ pairwise combinations, where $n_d$ is the number of replicate experiments at layer *d*, where $d=1$ indicates the top layer, $d=2,\ldots ,{D-1}$ indicate the intermediate layers and $d=D$ is the bottom layer in a study design.

In each replicate MS-Metabolomics experiment, a large number of unique metabolites with respect to mass-charge ratio and retention time are obtained. Each metabolite *m* examined is assumed to be associated with a continuous measurement from each of $n_D$ replicate experiments by the layer *D* (bottom layer), where *M* is the total number of metabolites. Let $x^{d}_{m,i}$ be the abundance measure of *m*th metabolite and the *i*th replicate experiment at layer *d*, such that, $m=1,2,\ldots ,M$ and $i=1,2,\ldots ,n_d$. We describe the structure and notation of these layers in the following sections. For notational simplicity, we will now use *I* as the total number of replicate experiments at the bottom layer.

#### Single layer

Let $x^{1}_{m,i}$ be the measure of *m*th metabolite and the *i*th replicate experiment under a single layered study design. An example of this study design is a data set that has the abundance measurements of *M* metabolites and *I* biological subjects (the replicate experiments in this case). See Fig. 2c for an example of a single-layered study design.

#### Double layer

Let $x^{\omega _1}_{m,i}$ be the measure of *m*th metabolite and the *i*th replicate experiment under a two-layered study design, i.e., $\omega _1$ (top layer) and *i* (bottom layer- replicate experiment level), where $\omega _1=1,\ldots ,n_{\omega _1}$ and $i=1,\ldots ,I$, such that, $n_{\omega _1}$ and *I* denote the total number of replicate experiments at the first (top) and bottom (second in this case) layers, respectively respectively respectively. An example of this study design is a data set that has abundance measurements of *M* metabolites and *I* replicate experiments under each of the $n_{\omega _1}$ biological subjects, i.e., in total there are $I \times n_{\omega _1}$ samples. See Fig. 2b for an example of a double-layered study design.

#### Triple layer

Let $x^{\omega _1,\omega _2}_{m,i}$ be the measure of *m*th metabolite and the *i*th replicate experiment under a three-layered study design, i.e., $\omega _1$ (top layer), $\omega _2$ (middle layer) and *i* (bottom layer- replicate experiment level), where $\omega _1=1,\ldots ,n_{\omega _1}$, $\omega _2=1,\ldots ,n_{\omega _2}$ and $i=1,\ldots ,I$, such that, $n_{\omega _1}, n_{\omega _2} \ge 2$. $n_{\omega _1}$, $n_{\omega _2}$, and *I* denote the total number of replicate experiments at the first (top), second and bottom (third in this case) layers, respectively respectively. An example of this study design is a data set that has abundance measurements of *M* metabolites and *I* replicate experiments under two layered study design where the middle layer is the technical layer (e.g., different operators or runs) with $n_{\omega _2}$ technical replicates and the top layer is subject layer with $n_{\omega _1}$ biological subjects, i.e., in total there are $I \times n_{\omega _1} \times n_{\omega _2}$ samples. See Fig. 2a for an example of a triple-layered study design.

#### *N* layer

Let $x^{\omega _1,\omega _2,\ldots ,\omega _{N-1}}_{m,i}$ be the measure of *m*th metabolite and the *i*th replicate experiment under an N-layered study design, i.e., $\omega _1$ (top layer), $\omega _2$ (second layer), $\ldots$, $\omega _{N-1}$ ($(N-1)$ layer) and *i* (bottom layer- replicate experiment level), where $\omega _1=1,\ldots ,n_{\omega _1}$, $\omega _2=1,\ldots ,n_{\omega _2}$, $\ldots$, $\omega _{N-1}=1,\ldots ,n_{\omega _{N-1}}$ and $i=1,\ldots ,I$, such that, $n_{\omega _1}, n_{\omega _2},\ldots ,n_{\omega _{N-1}} \ge 2$. $n_{\omega _1}$, $n_{\omega _2}$, $\ldots$, $n_{\omega _{N-1}}$ and *I* denote the total number of replicate experiments at the first (top), second, $\ldots$, $(N-1)$ and bottom (last) layers, respectively.

### MaRR procedure for MS-metabolomics

For notational simplicity, we assume only the single layered study design to describe the MS-Metabolomics data sets in the context of the MaRR procedure as discussed in “Single layer section”. MaRR assumes no missing values (see below for data pre-processing steps regarding missing values). Even though, we have data for more than two replicate experiments, the MaRR application to MS-based metabolomics data focuses on pairwise replicate experiments. Thus, under the single layered study design, we implement MaRR on pairwise combinations of *I* replicate experiments, i.e., ${I \atopwithdelims ()2}$ combinations of pairwise replicate experiments, where ${\mathbf {x}}_{i}$ and ${\mathbf {x}}_{i'}$ are the replicate data sets, such that, $i\ne i^{'}$, where ${\mathbf {x}}_{i}=(x_{1,i},\ldots ,x_{M,i})^{'}$. Moreover, ${\mathbf {X}}$ is a $M \times I$ matrix, such that, ${\mathbf {X}}=({\mathbf {x}}_{1},\ldots ,{\mathbf {x}}_{I})$, where ${\mathbf {x}}_{i'}$ be the vector of abundances of *M* metabolites on the *i*th replicate experiment. These abundances are converted into rank statistics. Each metabolite is assigned a rank in each of the two replicate: $(R_{m,i},\,R_{m,i^{'}})$, where $R_{m,i}$ is the rank among $x_{1,i},\ldots ,x_{M,i}$ and likewise for $R_{m,i^{'}}$. Now, the maximum rank statistic for the metabolite *m* can be defined as,

1 $$\begin{aligned} \hbox {Max}_{m}=\hbox {max}(R_{m,i},\,R_{m,i^{'}}), \end{aligned}$$

for $$m=1,\ldots ,M$$.

Consider Additional file 1: Table S1 detailing a subset of a real data of $M=6$ metabolites to describe the calculation of maximum rank statistics from a pair of replicate experiments. It is to be noted that metabolites that are highly ranked will have a relatively low value for their maximum rank statistic. On the other hand, low ranked metabolites will have higher values. Thus, choosing a threshold value based on the maximum rank can have the potential to separate reproducible from irreproducible signals.

#### Estimator of the proportion of reproducible metabolites

We define the proportion of reproducible metabolites between sample pairs as $\pi _1$. Due to the first assumption of the MaRR procedure under ideal settings [26], $\hbox {Max}_{y}<\hbox {Max}_{z}$ for all reproducible metabolites *y* and irreproducible metabolites *z*. This implies that all metabolites *y* such that $\hbox {Max}_{y}/M \le \pi _1$ are reproducible, and all metabolites *z* such that $\hbox {Max}_{z}/M >\pi _1$ are irreproducible. Rank pairs and maximum rank statistics for a sample data set generated under the ideal assumptions with $\pi _1 = 0.35$ are provided in Additional file 1: Fig. S1. MaRR uses the survival function in its estimator derivation of $\pi _1$. Thus, the empirical survival function is given by,

2 $$\begin{aligned} {\hat{S}}_{M}(x)= \frac{1}{M}\sum _{y=1}^{M}{I(\hbox {Max}_{y}/M\ge x)}, \quad x \in (0,1). \end{aligned}$$

Let $\pi _1 \in (0,1)$ be fixed, then according to the properties of MaRR under the ideal setting, the marginal limiting distribution of the random variable $\hbox {Max}_{z}/M$ as $M \rightarrow \infty$,

$$\begin{aligned} S_{\pi _1}(x)= {\left\{ \begin{array}{ll} 1 &{} x<\pi _1\\ 1-\frac{(x-\pi _1)^2}{(1-\pi _1)^2}, &{} \pi _1\le x \le 1. \\ 0 &{} 1<x \end{array}\right. } \end{aligned}$$

A weighted mean squared error between two functions for $\lambda \in (0,1)$ is defined as:

3 $$\begin{aligned} MSE_{M}(\lambda )=(M-l_{\lambda })^{-1}\sum _{x=l_{\lambda }}^{M}{[{\hat{S}}_{M}(x/M) - (1-\lambda )S_{\lambda }(x/M)]^2}, \end{aligned}$$

where $l_{\lambda }=\max _{l=1,\ldots ,M} (l: l/M \le \lambda )$. The minimizer of $MSE_{M}(\lambda )$ is used to estimate $\pi _1$. [26] showed the desirable performance of this estimator across a variety of scenarios.

We assume the following under the realistic setting:

- Reproducible signals tend to be ranked higher than irreproducible signals, that is, $P(R_{y,i} < R_{y,i^{'}})>1/2$ and $P(R_{z,i} < R_{z,i^{'}})>1/2$ if metabolite *y* is reproducible and metabolite *z* is irreproducible for any replicate sample pair $(i,i^{'})$.
- The correlation between the ranks of reproducible signals is nonnegative.
- The two ranks per irreproducible metabolite are independent.

The important difference between assumptions that separates the first assumption of realistic and ideal settings is the lack of clear split between reproducible and irreproducible signals with respect to $\hbox {Max}_{y}$. As a result, the estimator $\hat{\pi _1}$ derived in [26] is consistent in the ideal case whereas conservatively biased in the realistic case. In realistic settings, reproducible signals $\hbox {Max}_{y}/M$ have a positive probability of falling in the region $(\pi _1,1)$.

For computational convenience, the discrete and rescaled version of $\pi _1$ was used, i.e., ${\hat{k}}$.

4 $$\begin{aligned} {\hat{k}}= \underset{{l=l_{\lambda }}}{\text {arg min}}[MSE_{M}(l/M)], \end{aligned}$$

where $$l_{\lambda }=\displaystyle \max _{\{l=1,\ldots ,M \}} (l: l/M \le \lambda )$$.

In practice, ${\hat{k}}$ is a good estimate when reproducible signals begin transition to irreproducible signals. We chose the value of $\lambda$ to be 0.9 on the basis of a large number of simulated datasets with varying degrees of effect size and proportion of reproducible signals. However, this assertion cannot yet be proven theoretically. Although, for certain datasets with small effect size (e.g., mean parameter), we might need to reduce the $\lambda$ value [26]. In our MS-Metabolomics datasets, it was not required as the effect sizes were relatively large compared to the real RNA-seq datasets in [26].

#### Estimation of reproducible signal for metabolite *m* and replicate sample pair $(i,\,i')$

To determine the set of reproducible metabolites, a critical value ${\hat{N}}$ was chosen, according to an error rate. All metabolites from a replicate sample pair experiment $(i,i^{'})$ will be assigned reproducible if $\hbox {Max}_{y} \le {\hat{N}}$. This approach of declaring metabolites is defining a rejection as $(0,{\hat{N}})$, and rejecting the null hypothesis [irreproducibility for all signals with $\hbox {Max}_{y}$ in the region $(0,{\hat{N}})$] [27]. When an irreproducible metabolite was declared reproducible, Type I error (false discovery) was committed. Marginal false discovery rate (mFDR) is estimated based on a rejection region [28].

We assume *z* be the possible outcomes from simultaneous hypotheses [29], where *U* is the number of true null hypothesis that were correctly not rejected (true negatives), *V* is the number of false rejections (false positives), *T* is the number of hypotheses that were not rejected when they should have been (false negatives), and *S* is the number of correctly rejected hypotheses (true positives). *Q* is the total number of rejections made (rejected null hypotheses). The mFDR [28] is given by,

5 $$\begin{aligned} mFDR=\frac{E[V]}{E[Q]}. \end{aligned}$$

The above quantity is similar to the classical FDR as defined by [30]. To define mFDR estimate based on the MaRR procedure, the following notations are introduced:

$$\begin{aligned}&Q(l)=\sum _{y=1}^{M}I(\hbox {Max}_{y} \le l)= \text {Number of metabolites declared reproducible for critical region}\,(0,\,l).\\&V_k(l)=\text {Irreproducible metabolites declared reproducible with}\,k< \hbox {Max}_{y} \le l. \end{aligned}$$

By using the above notations, the mFDR estimate for using *l* as the threshold value for declaring reproducibility is given by,

6 $$\begin{aligned} m{\widehat{FDR}}(l)=\frac{E[V_{{\hat{k}}}(l)]}{Q(l)}. \end{aligned}$$

The denominator of the above expression can be directly computed from the data whereas the numerator needs to be calculated using the distribution of $\hbox {Max}_{z}$ and is also dependent on ${\hat{k}}$ [26]. The final expression of the numerator is given by,

7 $$\begin{aligned} E[V_{{\hat{k}}}(l)]= \frac{(l-{\hat{k}})^2}{M-{\hat{k}}},\quad l={\hat{k}}+1,\ldots ,M. \end{aligned}$$

The mFDR estimate associated with any rejection region $(0,\,l)$ can then be defined as,

8 $$\begin{aligned} m{\widehat{FDR}}(l)=\frac{E[V_{{\hat{k}}}(l)]}{Q(l)}=\frac{(l-{\hat{k}})^2}{Q(l)(M-{\hat{k}})},\quad l={\hat{k}}+1,\ldots ,M. \end{aligned}$$

We summarize the MaRR procedure for set of *m* metabolites each with two metrics generated from sample pair experiments as below:

9 $$\begin{aligned} {\hat{k}}= \underset{{l=l_{\lambda }}}{\text {arg min}}[MSE_{M}(l/M)], \end{aligned}$$

The FDR is controlled at a nominal level $\alpha$ if the threshold value ${\hat{N}}$ is chosen to be

10 $$\begin{aligned} {\hat{N}}=\displaystyle \max _{{\hat{k}} <l \le M} \{l:m{\widehat{FDR}}(l) \le \alpha \}, \end{aligned}$$

and the set of metabolites generated from replicate pair of experiments with maximum rank statistics less than or equal to ${\hat{N}}$ are declared reproducible. We detect the indices of the metabolites from replicate sample pair experiments which are declared reproducible. We define an indicator variable $r_{{m,(i,i')}}=1(0)$ to be a reproducible (irreproducible) signal for metabolite *m* and replicate sample pair $(i,i')$ as below:

$$\begin{aligned} r_{{m,(i,i')}}= {\left\{ \begin{array}{ll} 1, &{} I(\hbox {Max}_{m} \le {\hat{N}})\\ 0 &{} {\textit{otherwise}}. \end{array}\right. } \end{aligned}$$

### Evaluating reproducibility

We create a matrix of reproducible signals with *M* rows (total number of metabolites) and *J* columns ($J={I \atopwithdelims ()2}$) (replicate sample pairs ${I \atopwithdelims ()2}$), where *J* is the total possible number of sample pairs of replicate experiments. We assign metabolite *m* to be reproducible if a certain percentage signals ($100c_s\%$) are reproducible for pairwise combinations of replicate experiments across all study designs, i.e., if

11 $$\begin{aligned} \frac{{\sum _{i<i'}{{{r_{m,{(i,i')}}}}}}}{J} >c_s, \end{aligned}$$

such that, $$c_s \in (0,1)$$.

**Fig. 1:**
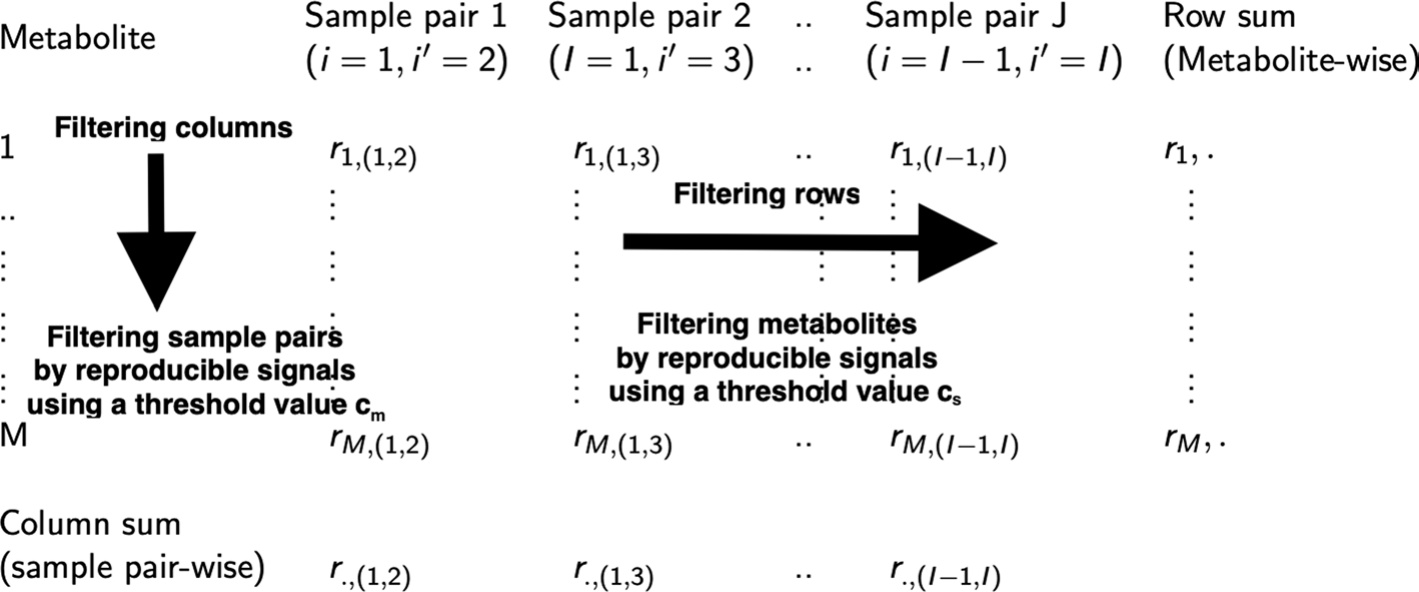
Schematic filtering approach of reproducible signal matrix. Schematic diagram showing the evaluation of MS-Metabolomics data. The schematic filtering approach can is data-adaptive because the filtering cut-offs ($c_s$ and $c_m$) can be specified to the filtering requirements of the data by the user

Similarly, we assign a sample pair $(i,i')$ for each study design ${\omega _1}$ to be reproducible if a certain percentage signals ($100c_m\%$) are reproducible across all metabolites, i.e., if

12 $$\begin{aligned} \frac{\sum _{m}{{r{_{m,(i,i')}}}}}{M}>c_m, \end{aligned}$$

such that, $c_m \in (0,1)$. Figure 1 illustrates the schematic filtering approach of reproducible signal matrix by rows (metabolites) and columns (sample pairs).

### Illustrative data sets

Chronic Obstructive Pulmonary Disease (COPD) is a major cause of morbidity and mortality in the United States. The multi-center Genetic Epidemiology of COPD (COPDGene) study was designed to study the underlying genetic factors of COPD, [31]. This study enrolled 10,263 individuals from 2008 to 2011 (Visit 1) who were aged 45–80 with $\ge 10$ pack-year smoking history and no exacerbations for $>30$ days. From 2013 to 2017, 6758 subjects returned for an in-person 5-year visit (Visit 2). Each in-person visit included spirometry before and after albuterol, quantitative CT imaging of the chest, and blood sampling.

#### Technical (Tech) data set

Three technicians performed all steps of sample prep for profiling of a single base human plasma sample collected from COPDGene visit 1 containing six spiked in control compounds at concentrations of 1X, 2X and 4X and two negative controls at 1X in all samples. Processing of the raw data was performed in Agilent’s Mass Hunter software.

**Fig. 2:**
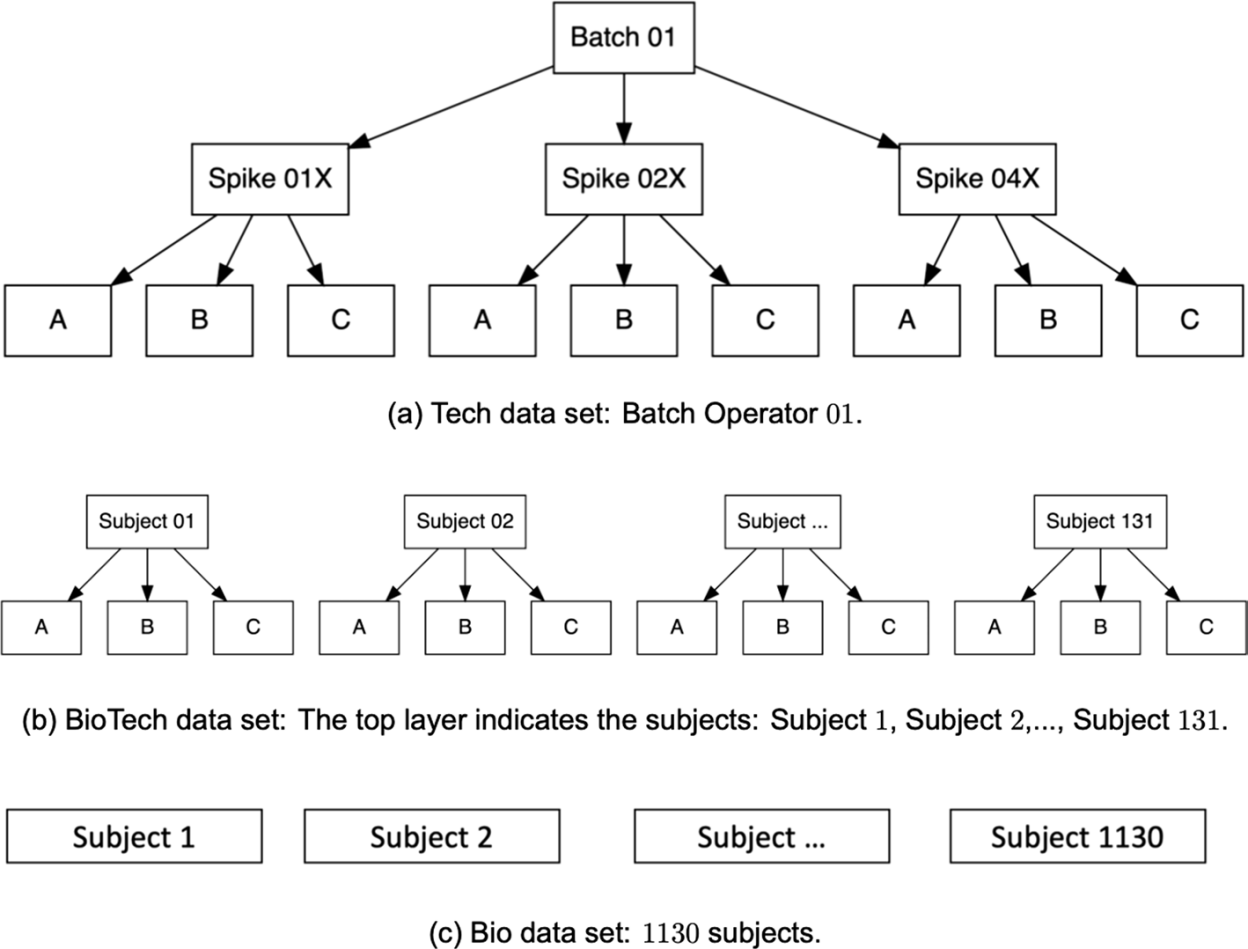
Flow charts of the 3 data sets. Flow charts show the hierarchical structure of the Tech, BioTech and Bio data sets. **a** The Tech data set is a triple-layered MS-Metabolomics experiment. **b** The BioTech data set is a double-layered MS-Metabolomics experiment. **c** The Bio data set is a single-layered MS-Metabolomics experiment

The Tech data set is a triple-layered MS-Metabolomics experiment. Figure 2a shows the hierarchical structure of the Tech data set, (i) the top layer being the batch operators, (ii) the middle layer is the spike-ins under each batch operator, and (iii) the bottom layer is the technical replicates under each spike-in. Based on our generic notations in “Triple layer section”, we can write, $n_{\omega _1}$, $n_{\omega _2}$ and *I* be the total number of replicate experiments at the top (batch operator), middle (spike-in control) and bottom (technical replicate) layers, respectively. Additional file 1: Table S2(a) illustrates the design of the MS-Metabolomics experiment for a single operator of the Tech data set.

#### Biological–Technical (BioTech) data set

Fresh frozen plasma was collected at COPDGene Visit 1 from 131 subjects. These samples were analyzed using untargeted LC–MS (C18+ and HILIC+) metabolomics. The lipid fraction of the human plasma collected from current and former smokers was analyzed using Time of Flight (ToF) liquid chromatograph (LC) (Agilent 6210 Series) and a Quadrupole ToF mass spectrometer (Agilent 6520) which yielded combined data on 2999 metabolite features before data-preprocessing. Data are available at the Metabolomics Workbench with Study ID ST000601, and data processing is described in [32].

The BioTech data set (Fig. 2b) can be treated as a double-layered MS-Metabolomics experiment as described in “Double layer section”. We can write, $n_{\omega _1}=131$ and $I=3$ be the total number of replicate experiments at the top (biological subjects) and bottom (technical replicates), respectively. Additional file 1: Table S2(b) illustrates the design of the MS-Metabolomics experiment for the BioTech data set.

#### Biological (Bio) data set

Within COPDGene 1136 subjects participated in an ancillary study in which they provided fresh frozen plasma at Visit 2. The plasma was profiled using the Metabolon Global Metabolomics platform using an untargeted gas chromatography–mass spectrometry and liquid chromatography–mass spectrometry (GC–MS and LC–MS) based metabolomic quantification protocol as described previously [12, 33]. The platform reported 1392 features including 1064 annotated features. A data normalization step was performed to correct variation resulting from instrument inter-day tuning differences: metabolite intensities were divided by the metabolite run day median then multiplied by the overall metabolite median. Subjects with aggregate metabolite median z-scores greater than 3.5 standard deviation from the mean $(\hbox {N}=6)$ of the cohort were removed.

The Bio data set (Fig. 2c) is a single-layered MS-Metabolomics experiment (“Single layer section”). Note that, there are no technical replicates in this data set. We can write, $x^{1}_{m,i}$ be the abundance measures of *m*th metabolite and the *i*th replicate experiment where $i=1,\ldots ,I(=1130)$. $I=1130$ denotes the total number of replicate experiments. Additional file 1: Table S2(c) illustrates the design of the MS-Metabolomics experiment for the Bio data set.

### Data pre-processing

We processed the three data sets described in “Illustrative data sets section” using the MSPrep software [34]. The data sets used in this paper are all log transformed. The data pre-processing include three steps and they are as follows:

#### Filtering

For each of the data sets, metabolites with more than 20% missing values were removed [22, 35]. (a) Tech data set-Post filtering, $M=860$ metabolites remain. (b) BioTech data set-Post filtering, $M=2860$ metabolites remain. (c) Bio data set-Post filtering, $M=995$ metabolites remain.

#### Missing value imputation techniques

For all data sets, we used BPCA to impute missing values, which implements a Bayesian version of the PCA (Principal Component Analysis) [36, 37]. It combines an Expectation Maximization (EM) approach for PCA with a Bayesian model and imputes the missing values. This R function is available in R/Bioconductor package pcaMethods. As a secondary analysis to compare imputation methods, we also used *k*NN and Random Forest (RF) imputations for the BioTech dataset. *k*NN imputation was originally developed for high-dimensional microarray gene expression data [38]. For each metabolite with missing values, this method finds the *k* ($=5$) nearest metabolites using Euclidean metric and missing values are imputed by averaging the nearest neighboring non-missing values. We applied the R package VIM for this imputation approach. Further, we used RF to impute the missing values. In many literatures, it has been observed that RF-based imputation outperformed other imputation methods for imputing metabolomics data [39–41]. We applied the R package missForest for RF-based imputation.

#### Normalization

Unwanted variation appears from various sources in metabolomics studies. Normalization is an important step for the downstream statistical analysis of metabolomics data. For all three data sets, we employ the msnormalize function with the quantile normalization options in MSPrep [34] to normalize the data while maintaining biological variation in the replicates. As a secondary analysis to compare normalization methods, we also used the median normalization option in MSPrep [34] for the BioTech data set.

### Pooling the abundance measurements

To evaluate the top layer of the hierarchy in the MS-metabolomics data design, we need to sum the abundances in the lower layer levels. We illustrate the sum of abundance measurements approach to measure reproducibility at each layer of the hierarchical MS-Metabolomics data sets using the Tech data set. For within spike-ins, i.e., between technical replicates, we rank and compare metabolites from each replicate pair. To make comparisons between spike-ins using the same operator, we sum abundance measurements over three replicates per spike-in. Subsequently, we rank the total abundance measurements for each spike-in and apply MaRR to measure the reproducibility metrics ($\pi _1$s) pairs of spike-ins under each batch operator, i.e., nine pairs of spike-in across three batch operators. Further, to make comparisons between batch operators for the same biological subject, we sum abundance measurements over all replicates under three spike-in per batch operator. MaRR is then applied to these pooled data set. We also repeat the sum of abundance measurements approach in the BioTech data set. However, this approach is not required to perform in the Bio data set (single-layered MS-Metabolomics data set).

### Dealing with ties

In the rare occurrence of ties, ties in the ranking method were treated by random assignment. For example, if there are 10 metabolites and 2 are tied for smallest values, then one of the tied metabolites will be randomly assigned to the 9th position and the other one to the 10th position.

### Simulations

The main goal of the simulation settings is to generate data with known reproducible/irreproducible signals that mimics the characteristics of one of our example data sets (BioTech described in “Illustrative data sets section”) to examine the performance of MaRR procedure. For this purpose, we describe two sets of simulation studies under extensive simulation settings. Since the MaRR procedure is applied to pair of replicate experiments, and to simplify notation, we assume one layer. In these simulations, we evaluate the accuracy of estimates of the reproducible signals $\pi _1$, FDR control and the power for detecting the true reproducible signals. In the simulations, we assume that the data are log transformed.

### Settings for simulation I

In simulation I, we generate data from a Bivariate Normal distribution using the means, standard deviations and Pearson’s correlation coefficient between replicate pair experiments of the processed BioTech data set.

We first ran the MaRR procedure to identify which metabolites were reproducible or irreproducible (see Methods), then calculated summary statistics of the reproducible/irreproducible metabolites and used those to generate simulation data sets In summary, (i) the quartiles of the proportion of reproducible signals ($\pi _1$) ranged between 0.90 and 0.95 for 393 sample pairs and 2860 metabolites. (ii) The quartiles of the means of the reproducible abundance measures ($\mu _R$), for replicate experiments ranged between 3.89 and 4.13. (iii) The quartiles of the means of the irreproducible abundance measures ($\mu _{IR}$), for replicate experiments ranged between 3.19 and 3.21. (iv) The quartiles of the standard deviations of the reproducible abundance measures ($\sigma _R$) for replicate experiments ranged between 0.16 and 0.17. (v) The quartiles of the standard deviations of the irreproducible abundance measures ($\sigma _{IR}$) for replicate experiments ranged between 0.04 and 0.05. (vi) The quartiles of the Pearson’s correlation coefficient of the reproducible abundance measures ($\rho _R$) between replicate experiments ranged between 0.992 and 0.994. We summarized the quartile measurements of these parameters in Additional file 1: Table S3. In addition, we presented the boxplots showing the spread of these parameters in Additional file 1: Fig. S2.

We fix the true values of the following parameters because their quartile ranges were extremely narrow: mean of the irreproducible abundance measures for replicate experiments ($\mu _{IR}=3.2$), standard deviation of the reproducible abundance measures for replicate experiments ($\sigma _R=0.17$) and standard deviation of the irreproducible abundance measures for replicate experiments ($\sigma _{IR}=0.05$). For the other parameters, we chose values from the quartile ranges: the mean of the reproducible abundance measures ($\mu _R$), the proportion of reproducible signals ($\pi _1$), and the correlation between reproducible pairs of abundance measures($\rho _R$).

Under realistic settings of the MaRR procedure, (i) we assume that test statistics with large values will be highly ranked and (ii) the correlation between irreproducible abundance measure is zero. The test statistics for reproducible signals are generated from the Bivariate Normal distribution as follows:

$$\begin{aligned} \left( \begin{array}{c} t_{m,1}\\ t_{m,2} \end{array}\right)\sim & {} {\mathcal {N}} \left[ \left( \begin{array}{c} \mu _R\\ \mu _R \end{array}\right) ,\left( \begin{array}{cc} \sigma _R^2 &{} \sigma _R^2 \rho _R\\ \sigma _R^2 \rho _R &{} \sigma _R^2 \end{array}\right) \right] . \end{aligned}$$

The test statistics for irreproducible signals are generated from the standard bivariate Normal distribution assuming that the two test statistics are independent:

$$\begin{aligned} \left( \begin{array}{c} t_{m,1}\\ t_{m,2} \end{array}\right)\sim & {} {\mathcal {N}} \left[ \left( \begin{array}{c} \mu _{IR}\\ \mu _{IR} \end{array}\right) ,\left( \begin{array}{cc} \sigma _{IR}^2 &{} 0\\ 0 &{} \sigma _{IR}^2 \end{array}\right) \right] . \end{aligned}$$

To make the situations more challenging and exhaustive, we include 24 settings by varying $\mu _R\in \{3.89,4.01,4.13\}$, $\rho _R\in \{0.45,0.99\}$, and $\pi _1\in \{0.2,0.4,0.75,0.9\}$ based on the summary statistics described above For each setting, we simulate 1000 data sets of metabolite size $M=2860$, which is the number of metabolites in the BioTech data set, and apply the MaRR procedure.

### Settings for simulation II

The simulation design is kept similar to the original MaRR paper [26]. Thus, to test the performance of the MaRR under extensive simulation settings, we implement the simulations with extreme choices of the parameters ($\pi _1$ and $r_0$), and do not assume normality for all values.

Each simulated data will consist of proportion of reproducible signals and a minimum correlation for lowest ranked signals as parameters. The largest values of $t_{m,1}$ and $t_{m,2}$ are assumed to be highly ranked metabolites. For each reproducible metabolite *m*, the first test statistic $t_{m,1}$ is generated according to $t_{m,1} \sim {\textit{Uniform}}(4,5)$. The second test statistic $t_{m,2}$ is dependent in such a way that, the correlation between $t_{m,1}$ and $t_{m,2}$ is linearly dependent according to a Normal distribution given $t_{m,1}$,

13 $$\begin{aligned} t_{m,2}| t_{m,1} \sim {\mathcal {N}}(t_{m,1},1-r_m^{2}), \end{aligned}$$

where $$r_m=\frac{1-r_0}{5-4}(t_{m,1}-4)+r_0$$.

For the correlation structure, we assume that when $t_{m,1}=5$, there is perfect correlation whereas, the minimum correlation for the lowest ranked reproducible signals ($t_{m,1}=4$) is $r_0$.

As with Simulation I, the test statistics for irreproducible metabolites are generated from the bivariate Normal distribution assuming that the two test statistics are independent,

$$\begin{aligned} \left( \begin{array}{c} t_{m,1}\\ t_{m,2} \end{array}\right)\sim & {} {\mathcal {N}} \left[ \left( \begin{array}{c} \mu _{IR}\\ \mu _{IR} \end{array}\right) ,\left( \begin{array}{cc} \sigma _{IR}^2 &{} 0\\ 0 &{} \sigma _{IR}^2 \end{array}\right) \right] . \end{aligned}$$

We consider 12 parameter settings for this simulation design by varying the proportion of reproducible signals, $\pi _1\,\in \,\{0.2,\,0.4,\,0.75,\,0.9\}$ and the minimum correlations $r_0\,\in \,\{0.4,\,0.6,\,0.99\}$ as selected above. For each setting, we simulate 1000 data sets and apply the MaRR procedure. To imitate the MS-Metabolomics data more closely for each data set, the size of metabolites is chosen to be $M=2860$, which is the number of metabolites for the BioTech data set.

For each simulated data set, we compute the empirical FDR based on MaRR independently by dividing the number of false positives by the total number of reproducible signals. Similarly, we also compute the empirical NDR by dividing the true negatives by the total number of true reproducible signals.

### Settings for simulation III

This simulation design is kept exactly similar to the settings for Simulation I except the test statistics for irreproducible signals are generated from the standard bivariate t distribution with degrees of freedom 3 assuming that the two test statistics are independent. The Standard t-distribution with degrees of freedom has heavier tails compared to Standard Normal distribution.

## Results

### Simulation results

We assess the performance of the MaRR procedure using simulated data sets designed to resemble the output from MS-Metabolomics experiments for the three sets of simulation studies.

#### FDR control

**Fig. 3:**
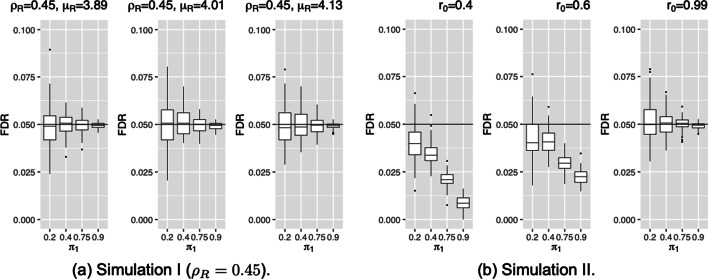
FDRs for simulations I and II based on 1000 simulated datasets in each setting. The horizontal line indicates the target FDR level ($\alpha =0.05$) for all simulations. Labels along the x-axis describe values of $\pi _1$

Figure 3 and Additional file 1: Fig. S3 compare the FDR control for $\rho _R=0.45$ of Simulation I. The FDR results illustrate that the MaRR procedure effectively controls FDR across all settings of Simulation I at 5% level of significance. The FDR results across all choice of $\mu _R$ show that the variability is increasing as $\pi _1$ decreases. This was expected as the MaRR procedure performs best when the proportions of reproducible signals are high.

In Simulation II (Fig. 3), the FDR results demonstrate that the MaRR procedure performs well in controlling the FDR in almost all situations. Here also, we observe that the MaRR procedure can be very conservative when the proportion of reproducible signals are high and $r_0$ are low. A downward trend can be observed with increasing values of $\pi _1$.

#### Discriminative power

**Fig. 4:**
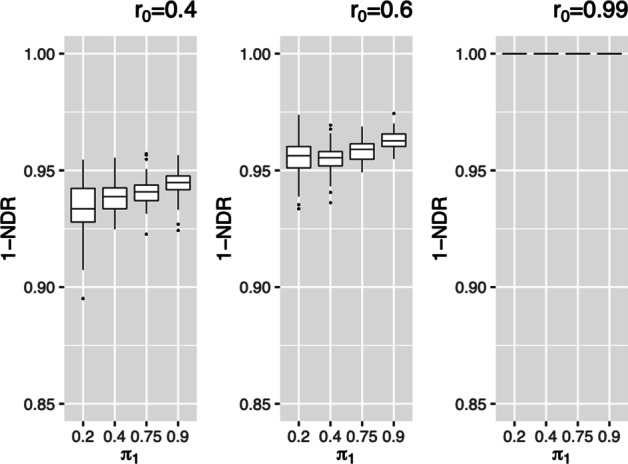
$1-NDR$ for simulation II based on 1000 simulated datasets in each setting. Labels along the x-axis describe values of $\pi _1$

We compare the discriminative power (1-NDR) for the same settings discussed above, where NDR is the Non-Discovery Rate (Type II error) [26]. The discriminative power of simulations I ($\rho _R=0.45,\,0.99$) are almost 100% (Additional file 1: Figs. S4 and S5). For simulations II (Fig. 4), we also observe high discriminative power. The MaRR procedure has very high power for all simulation settings across I and II.

#### Bias of $\pi _1$

Additional file 1: Figs. S6 and S7 show the bias of the proportions of reproducible signals, i.e., $\pi _1$. The bias values across all simulations lie within a narrow range $(-0.05,\,0.05)$ indicating that the MaRR procedure performs sufficiently well in estimating $\pi _1$. In Simulation I, the bias is positive and monotonically increasing with the increment of $\pi _1$ irrespective of the choices of $\rho _R$ and $\mu _R$. Additional figures when $\rho _R=0.99$ are presented in Additional file 1: Fig. S6. In Simulation II, the bias is negative and monotonically decreasing with the decrease of $\pi _1$ except when $r_0=0.99$. For $r_0=0.99$, the bias behaves similar to Simulation I.

Simulation III results were similar to Simulation I. We presented the results in the Additional file 1: Figs. S8–S13. Further, we also compared the performance of the MaRR procedure and RSD. We find that the performance of the MaRR procedure is better compared to RSD. We provided the details about the simulation settings and results in the Additional file 1: Figs. S14 and S15.

### COPDGene data analysis

We apply the MaRR procedure to study the reproducibility of biological and technical replicates between and within study designs (e.g., operators, biological subjects) on real data sets. As described in previous section (“MS-metabolomics data design section”), MS-Metabolomics data can have multiple layers.

We applied the MaRR procedure to each of the three MS-Metabolomics data sets. The reproducible signals were identified for all three layers (batch, spike-in and replicates) in Tech data set; two layers (biological subjects and replicates) in BioTech data set and a single layer (biological replicates) in Bio data set. We declared a signal to be reproducible at an error controlling rate of $\alpha =0.01$ using the MaRR procedure. We assign a metabolite for a replicate sample pair and a sample pair for a metabolite to be reproducible using a threshold value of $c_s$ and $c_m$, respectively defined in “Evaluating reproducibility section”, and vary these values.

The distribution of reproducible pairs and metabolites are illustrated in Additional file 1: Figs. S16 and 5 for the Tech data set and Additional file 1: Figs. S17 and 6 for the BioTech and Bio data sets, respectively.

We label a metabolite and a sample pair to be reproducible if at least $100\%c_s$ and $100\%c_m$ of signals are reproducible across sample pairs of experiments and metabolites, respectively. We provide the percentage of reproducible metabolites and sample pairs based on the threshold value of greater than the percent threshold values of reproducible signals for the three data sets. The results of all the three data sets are also summarized in Table 1. For different values of $c_s$ and $c_m$, the entries in the table represent the proportion of sample pairs of replicate experiments with reproducible signals per sample pair a and proportion of metabolites with reproducible signals per metabolite, respectively. It is a more conservative approach to have higher percentage of threshold as it would filter higher number of metabolites or sample pairs.

#### Tech Data- Reproducibility of metabolites per sample pair

**Fig. 5:**
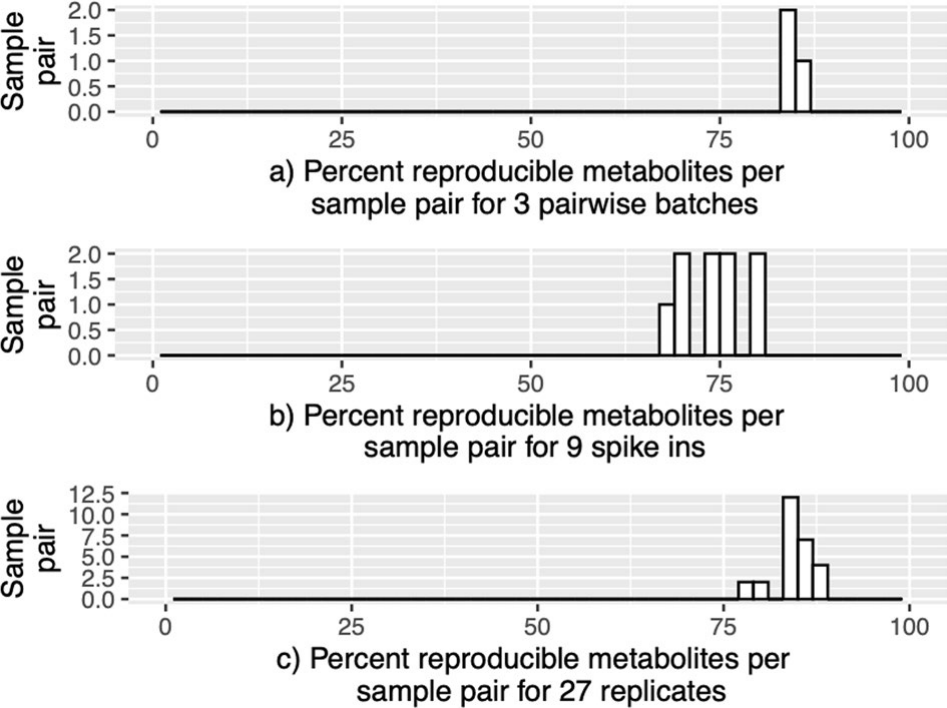
Histograms showing reproducible metabolites for three layers. These histograms are in percent scale where the *x*-axes denote the percent scale of reproducible metabolites for all possible sample pairs. **a** Top layer (batches): three pairs of batches. **b** Middle layer (spike-ins): nine pairs of spike-ins. **c** Bottom layer (technical replicates): 27 pairs of technical replicates

Figure 5 illustrates percentage of reproducible metabolites per sample pair in the *x*-axis. In Fig. 5, the higher percentage of reproducible metabolites per sample pair in the *x*-axis would indicate stronger reproducibility between the two samples (sample pairs), e.g., the percent metabolites in the last right bin indicates that all or almost all the metabolites are reproducible for the number of sample pair combinations in the *y*-axis. The percentage of reproducible metabolites per sample pair (close to 100%) are much higher for the Operator and technical layer compared to the spike-in layer data (Table 1).

#### Tech data-reproducibility of sample pairs per metabolite

Additional file 1: Fig. S16 illustrates percentage of reproducible sample pairs per metabolite in the *x*-axis. In all three layers (Additional file 1: Fig. S16a–c), the higher percentage would indicate strong reproducibility of a metabolite across all sample pairs for each of these layers. This indicates that there is high reproducibility of sample pairs per metabolite in all these three layers of technical replicates. Table 1 shows that the percentage of reproducible sample pairs per metabolite greater than different thresholds (70%, 80%, 90%) are more or less same, i.e., around 82%.

**Table 1:** Summary of reproducible metabolites per sample pair and reproducible sample pairs per metabolite for Tech, BioTech and Bio data sets

Data set		Percentage of reproducible metabolites per sample pair			Percentage of reproducible sample pairs per metabolite		
		> 70%	> 80%	> 90%	> 70%	> 80%	> 90%
Tech	Operator layer	100.00	100.00	0.00	82.09	82.09	82.09
	Spike-in layer	88.89	11.11	0.00	81.28	81.05	80.23
	Replicate layer	100.00	92.59	0.00	85.23	84.30	83.02
BioTech	Biological layer	49.85	9.59	0.73	64.44	60.80	55.07
	Technical layer	90.08	0.76	0.25	68.29	63.78	59.09
Bio	Biological layer	98.52	92.40	76.95	88.84	84.32	76.78

The columns of proportion of reproducible metabolites per sample pair $>70/80/90\%$ indicate that proportion of sample pairs of replicate experiments with reproducible metabolites per sample pair greater than $70/80/90\%$. The columns of proportion of reproducible sample pairs per metabolite $>70/80/90\%$ indicate that proportion of metabolites with reproducible sample pairs per metabolite greater than $>70/80/90\%$

#### BioTech and Bio data-reproducibility of metabolites per sample pair

**Fig. 6:**
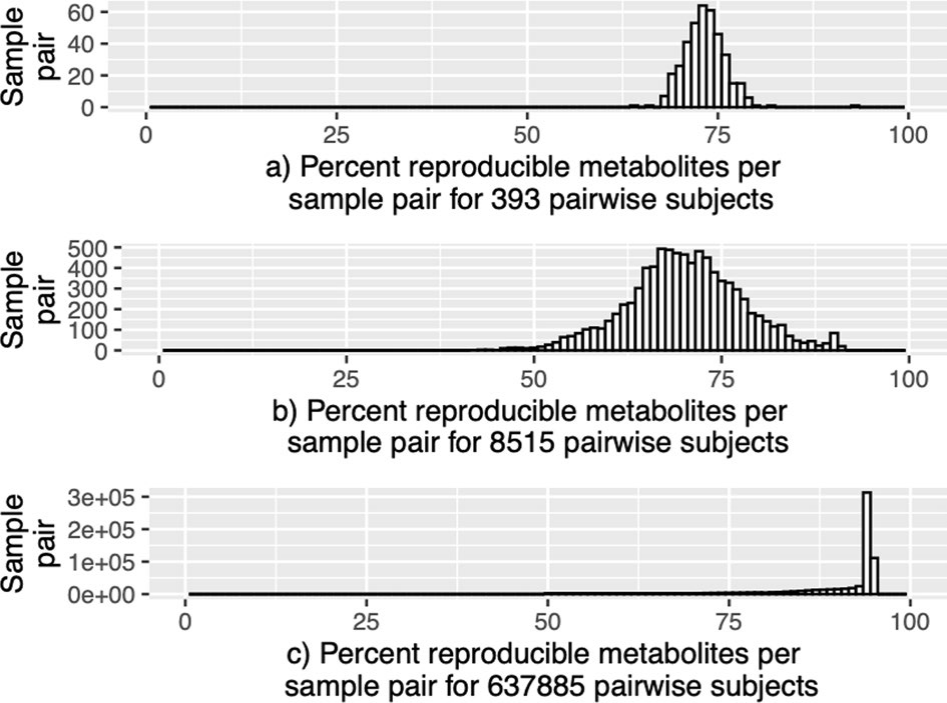
Histograms showing reproducible metabolites per sample pair for BioTech and Bio data sets. These histograms are in percent scale where the *x*-axes denote the percent scale of reproducible metabolites. Reproducible metabolites **a** 393 technical replicate sample pairs (BioTech), **b** 8515 biological replicate sample pairs (BioTech), and **c** 637,885 biological replicate sample pairs (Bio)

Figure 6 illustrates that the reproducibility of metabolites per sample pair are higher for technical replicates of BioTech data set (Fig. 6a) compared to biological replicates of the same data set (BioTech) (Fig. 6b). Table 1 also confirms the reproducibility of metabolites per sample pair is higher for technical replicates compared to its biological data set (BioTech). In BioTech data set, the percentage of reproducible metabolites per sample pair greater than 70% for technical replicates is 90.08% compared to 49.85% of biological replicates. In Fig. 6b and c, we also report that the reproducibility of metabolites per sample pair is much higher for Bio data set compared the biological replicates of Biotech data set. However, there are differences between the two technologies for BioTech and Bio data sets since the samples were not collected on the same subjects at the same visit for a direct comparison.

#### BioTech and bio data-reproducibility of sample pairs per metabolite

Additional file 1: Fig. S17 compares the percentage reproducible sample pairs per metabolite either across technical replicates (Additional file 1: Fig. S9a) or biological replicates (Additional file 1: Fig. S17b, c). However, in this case, we observe that the percentage of reproducible sample pairs per metabolite $>70\%$ threshold ($=68.29\%$) for technical replicates of the BioTech data set (bottom layer) (Additional file 1: Fig. S17a) is higher than their biological replicates (BioTech data set (top layer)) ($=64.44\%$) (Additional file 1: Fig. S17b) and lower than biological replicates ($=88.84\%$) of Bio data set (Fig. S17c), (Table 1). Also, note that, the samples were not collected on the same subjects at the same visit for a direct comparison.

#### Using MaRR to compare data processing methods

In the previous sections, we only presented MaRR reproducibility results with BPCA imputed and quantile normalized data. Here, we also illustrate how MaRR can be used to compare different combinations of imputation and normalization steps based on the resulting reproducibility of metabolites and sample pairs: we compare four combinations of imputation and normalization methods: (i) BPCA and quantile (as above), (ii) BPCA and median, (iii) kNN and median, (iv) kNN and quantile, (v) RF and median, (vi) RF and quantile. The results of all the BioTech dataset are summarized in Table 2. For the BioTech dataset, processing the data using RF-median or RF-quantile achieved the maximum reproducibility (Table 2). The additional figures (Additional file 1: Figs. S18–S23) for the BioTech and Bio data sets are provided using the MaRR procedure to compare data processing methods in the Additional file.

**Table 2:** Summary of reproducible metabolites per sample pair and reproducible sample pairs per metabolite for BioTech data set

Imputation	Normalization		Percentage of reproducible metabolites per sample pair			Percentage of reproducible sample pairs per metabolite		
			> 70%	> 80%	> 90%	> 70%	> 80%	> 90%
BPCA	Median	Biological layer	52.27	10.68	0.75	64.69	61.22	55.56
		Technical layer	90.08	0.76	0.25	68.29	63.78	59.09
	Quantile	Biological layer	49.85	9.59	0.73	64.44	60.80	55.07
		Technical layer	90.08	0.76	0.25	68.29	63.78	59.09
KNN	Median	Biological layer	14.14	0.47	0.00	56.01	52.41	47.06
		Technical layer	0.00	0.00	0.00	46.05	41.89	35.45
	Quantile	Biological layer	13.39	0.36	0.00	55.66	51.75	46.89
		Technical layer	0.00	0.00	0.00	46.05	41.89	35.45
RF	Median	Biological layer	75.30	30.85	3.73	69.62	65.87	60.77
		Technical layer	98.73	7.63	0.51	71.05	66.89	62.03
	Quantile	Biological layer	74.15	29.07	3.58	69.41	65.52	60.38
		Technical layer	98.73	7.12	0.51	71.12	67.03	62.03

The columns of proportion of reproducible metabolites per sample pair $>70/80/90\%$ indicate that proportion of sample pairs of replicate experiments with reproducible metabolites per sample pair greater than $>70/80/90\%$. The columns of proportion of reproducible sample pairs per metabolite $>70/80/90\%$ indicate that proportion of metabolites with reproducible sample pairs per metabolite greater than $>70/80/90\%$

#### Reproducibility comparisons using MaRR and RSD

We compared MaRR and RSD measurements in terms of reproducibility for the BioTech data set. The reason we chose BioTech data set since it is a combination of both biological and technical replicates. We examined reproducibility, in particular RSD measurements versus MaRR based reproducible (and irreproducible) metabolite index in a scatter plot (technical and biological replicates of BioTech data set using RF-quantile pre-processing). We assigned a metabolite to be reproducible if the percent of reproducible sample pairs per metabolite is greater than a particular threshold value ($75\%$) (Additional file 1: Figs. S24(a) and S24(b) for reproducible metabolites; Additional file 1: Fig. S24(c) and S24(d) for irreproducible metabolites). The RSD values in the y-axis are shifted slightly higher for irreproducible metabolites compared to reproducible metabolites. One would expect reproducible metabolites to have relatively high variability (RSD) across subjects (biological replicates) and low variability across replicate samples (technical replicates), which was clearly not the case. Generally, in analytical metabolomics research, the RSD is calculated across pooled Quality Control (QC) samples for each metabolite and those with an RSD above a pre-determined cutoff (e.g., 20–30%) are removed [5, 22, 24, 25, 42]. However, we find that the RSD is not an ideal predictor of identifying high quality (reproducible) metabolites (Additional file 1: Fig. S25 and Table S4-complementary to Fig. S24). We used a filtering cutoff of 25% (horizontal black line in Additional file 1: Fig. S25) but the cutoff does not remove any of the irreproducible metabolites. Even though it assesses variability across technical replicates relatively well, it fails to capture the true variability (separating the reproducible and irreproducible metabolites) across biological replicates. Our MaRR procedure can detect reproducible and irreproducible metabolites with greater accuracy as shown using simulations. Our findings about the predictive ability of RSD in identifying reproducible and irreproducible metabolites are also in concordance with the findings of low and high quality metabolites (features) shown in another approach of filtering procedures for MS-Metabolomics data [23].

#### Data-driven Reproducibility pipeline using MaRR

**Fig. 7:**
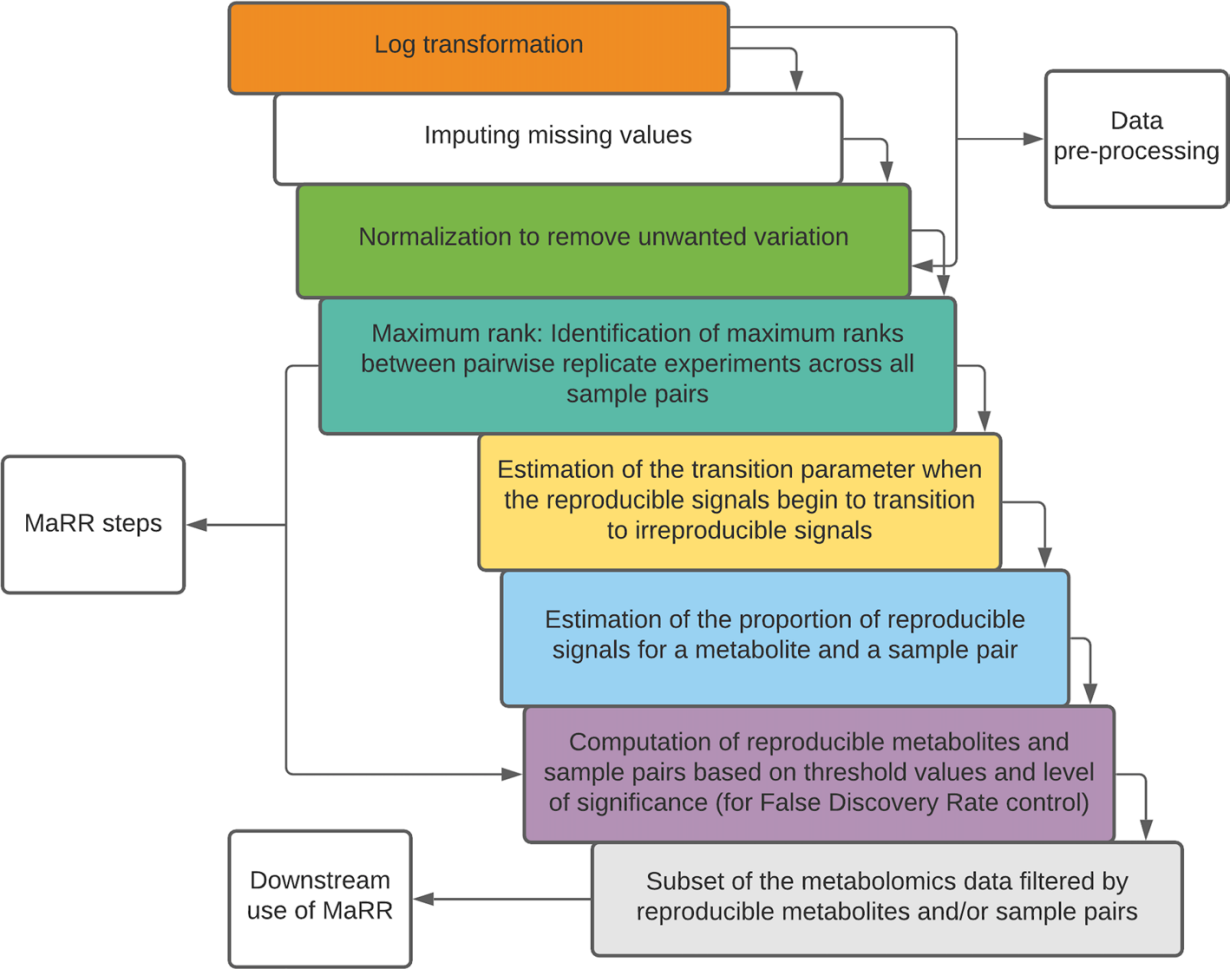
Data-driven reproducibility flow chart using the MaRR procedure. Flow chart of a data-driven reproducibility pipeline for MS-Metabolomics data. The approach is data-driven because the filtering cutoffs and level of significance can be specified by the user to identify reproducible metabolites and sample pairs prior to further downstream analysis

We argue that reproducibility methods should be data-driven and should not depend on a particular cutoff (or a range of cutoff, e.g., 20–30%). A data-driven pipeline is one which curates reproducibility to the specific requirements of a particular data set, rather than using predefined cutoffs. Here, we present a sequence of steps (Fig. 7) representing a data-driven pipeline in assessing reproducibility of MS-Metabolomics data. Our data-driven filtering approach consists steps for data pre-processing, identifying reproducible metabolites and sample pairs using the MaRR procedure and subsetting the reproducible data.

## Discussion

The biggest strength of MaRR is that with very limited assumptions, it can detect the reproducible signals efficiently. In the simulation studies, there were situations where the MaRR procedure was not conservative enough even though it achieved the main purpose of controlling the FDR at $\alpha =0.05$. This is due to the lack of separation between the reproducible and irreproducible components. In these scenarios, the real signals might fall in the undetectable regions because of overlapping between the reproducible and irreproducible components [43]. For smaller values of $\pi _1$, the finite sample performance deteriorates, making the detection of true signals unreliable. The powers for most of the situations were nearly one, meaning that all the true reproducible signals were discovered using the MaRR procedure. This indicates that the true reproducible signals identified using the MaRR procedure was high but these situations do not have inflated discriminative powers. Another interesting finding from the bias results was the systematic underestimation of $\pi _1$ using the MaRR procedure. This suggests that the MaRR assumption yield a conservative estimator of $\pi _1$. In summary, FDR controls error rates well, has high power. The simulations confirm that the MaRR procedure performs sufficiently well in most situations except for smaller values of $\pi _1$ and/or when the reproducible and irreproducible components are overlapping.

We applied the MaRR procedure to measure reproducibility both within and across batches using technical replicates for the Tech and BioTech datasets and biological replicates for the BioTech and Bio dataset. The MaRR procedure was originally developed to assess reproducibility of a pair of technical replicates (within labs and across labs variation) [26]. The MaRR procedure tends to favor highly expressed abundance measurements. We extended the idea to pairwise combinations of multiple replicate experiments for both technical and biological replicate experiments. Our method does not require to have prior knowledge of the proportion of reproducible signals. We have shown in our simulation studies that our method can work well with values of proportion of reproducible signals as low as 0.2 and as high as 0.99.

We compared the MaRR procedure to commonly used reproducibility method using the BioTech dat set. To compare the methods, we identified reproducible metabolites using the MaRR procedure and then computed RSD for both biological and technical replicates in the BioTech data set. Our results indicate that reproducibility score is not strongly associated with RSD. We also showed how our MaRR based data-driven filtering approach has the ability to remove irreproducible metabolites and sample pairs while retaining reproducible metabolites and sample pairs based on simulations.

For the real data sets, we estimated the reproducible sample pairs per metabolite as well as reproducible metabolites per sample pair. The estimated proportion of reproducible signals were considerably high. For MS-Metabolomics real data, we adopted a more conservative approach to identify reproducible signals, i.e., with an FDR control at $\alpha =0.01$ instead of $\alpha =0.05$. In the Tech data set (Fig. 5), the reproducible metabolites across all sample pairs (for operator layer and technical layer) were greater than 70%, indicating very high reproducibility (Table 1). For the BioTech data set (Fig. 6), the percentage of reproducible metabolites per sample pair greater than 70% for technical replicates is much higher compared to their biological replicates. This result has also confirmed that there is higher reproducibility for technical replicates compared to their biological replicates from the same data set (BioTech). Similarly, the reproducible metabolites almost across all biological sample pairs $(\sim 98.52\%)$ were greater than 70% threshold (Table 1). This could be due to false identification of true reproducible signals as irreproducible when there is a strong biological difference for a metabolite across samples. In addition, to create data-dependent threshold values ($c_s$ and $c_m$) in the MaRR steps of the pipeline (Fig. 7), we also allow the users to visualize the histograms (e.g., Figs. 5 and 6) and subsequently choose the more appropriate threshold values.

In this paper, our goal was not to do a comprehensive evaluation of pre-processing steps, but instead to show how this measure can be used as a benchmark for comparison. We used the MaRR procedure to compare different pre-processing pipelines. Our results simultaneously provide a useful assessment of three different replicate data sets (Tech, BioTech and Bio) in terms of reproducibility. Furthermore, our MaRR-based approach also allows the users to select the filtering threshold values ($c_s,\,c_m$) for the identification of reproducible metabolites and sample pairs based on thorough output visualization checks (histograms of percent reproducible metabolites per sample pair and percent reproducible sample pairs per metabolite).

## Conclusion

In this paper, we have developed a data-driven approach to select reproducible metabolites and sample pairs as a post-processing step to performing further downstream analysis. The MaRR procedure can be applied to metabolomics studies to primarily assess the reproducibility of MS-Metabolomics data sets. In addition, the other potential use of the MaRR procedure could be flagging metabolites and samples that are less reproducible. As illustrated in this paper, the MaRR procedure can also be employed to evaluate different pre-processing pipelines (e.g., missing value imputation, normalization).

This research was motivated by reproducibility, which has proven to be a major obstacle in the use of genomic findings to advance clinical practice [44]. We are hoping that this research will stimulate other groups to develop and evaluate new statistical frameworks for reproducibility.

## Supplementary Information

**Additional file 1**. Supplementary materials. Reproducibility of mass spectrometry based metabolomics data.

## Data Availability

Data files for all benchmark data sets are available from Metabolomics Workbench at https://www.metabolomicsworkbench.org with Project ID PR000438 (BioTech data set) and Project ID PR000907 (Bio data set). The Bio data set can be accessed directly via its Project DOI: 10.21228/M8FQ2X. The BioTech data set can be accessed directly via its Project DOI: 10.21228/M8FC7C. This work is supported by NIH grant, U2C DK119886. The methods described in this paper are implemented in the open-source R package *marr*, which is freely available from Bioconductor at http://bioconductor.org/packages/marr [45]. The *marr* package includes comprehensive help files for each function, as well as a package vignette demonstrating a complete example study design. A flowchart of the *marr* Bioconductor package is presented in the Additional file 1: Fig. S26. Code scripts to reproduce all data preparation, simulation steps, generate all figures and tables are available from GitHub at https://github.com/Ghoshlab/marr_paper_evaluations. The development version of the *marr* R package is available from GitHub at https://github.com/Ghoshlab/marr. In terms of computational time, on a desktop with a 3.2 GHz processor and 16 GB memory, marr takes approximately 12 min 47 s to analyze a MS-Metabolomics data with 662 metabolites (features) and ${393\atopwithdelims ()2}$ (i.e., 77028) sample pairs. marr takes approximately 13 min 02 s to analyze a MS-Metabolomics data with 2860 metabolites (features) and ${393\atopwithdelims ()2}$ (i.e., 77028) sample pairs. The computational complexity mainly depends on the sample pairs not on the number of metabolites (features). We have developed a Shiny-based Web application, called marr Shiny, for dynamic interaction with MS-metabolomics data that can run on any Web browser and requires no prior programming knowledge [https://maxmcgrath.shinyapps.io/marr/]. Illustrative screenshots of the marr Shiny app pipeline are in the Additional file 1: Figs. S27 and S28.
