# Supplementary material for: Reproducibility of mass spectrometry based metabolomics data
Source: BMC Bioinformatics. 2021 Sep 7;22:423. doi: 10.1186/s12859-021-04336-9 (PMC8424977; doi:10.1186/s12859-021-04336-9)
Supplement: Supplementary file 1 — Additional file 1. Supplementary materials. Reproducibility of mass spectrometry based metabolomics data. [file 12859_2021_4336_MOESM1_ESM.pdf]

Supplementary materials for the paper titled **Reproducibility of Mass Spectrometry based Metabolomics Data**

Tusharkanti Ghosh, Daisy Philtron, Weiming Zhang, Katerina Kechris and Debashis Ghosh

1 Maximum rank statistics from six metabolites

Index (m)	$\mathbf{x}_i$	$\mathbf{x}_{i'}$	$(R_{m,i},\ R_{m,i'})$	Max_m
1	3.87	3.92	(6,5)	6
2	5.73	5.54	(3,3)	3
3	4.19	3.89	(5,6)	6
4	6.62	6.59	(2,2)	2
5	8.70	8.53	(1,1)	1
6	5.15	5.09	(4,4)	4

Table S1: Sample data ranks and maximum rank statistics from six metabolites in a replicate experiments (BioTech data set). Table detailing a subset of a real data of $M = 6$ metabolites to describe the calculation of maximum rank statistics from a pair of replicate experiments. Note, metabolites that are highly ranked will have a relatively low value for their maximum rank statistic. On the other hand, low ranked metabolites will have higher values. Thus, choosing a threshold value based on the maximum rank can have the potential to separate reproducible from irreproducible signals.

2 Data Structure of three data sets

	Batch Operator A								
Metabolite	Spike-in 01X			Spike-in 02X			Spike -in 04X		
	Replicate 1	Replicate 2	Replicate 3	Replicate 1	Replicate 2	Replicate 3	Replicate 1	Replicate 2	Replicate 3
1	$x_{1,1}^{1,1}$	$x_{1,2}^{1,1}$	$x_{1,3}^{1,1}$	$x_{1,1}^{1,2}$	$x_{1,2}^{1,2}$	$x_{1,3}^{1,2}$	$x_{1,1}^{1,3}$	$x_{1,2}^{1,3}$	$x_{1,3}^{1,3}$
. . $\vdots$	$\vdots$	$\vdots$	$\vdots$	$\vdots$	$\vdots$	$\vdots$	$\vdots$	$\vdots$	$\vdots$
M	$x_{M,1}^{1,1}$	$x_{M,2}^{1,1}$	$x_{M,3}^{1,1}$	$x_{M,1}^{1,2}$	$x_{M,2}^{1,2}$	$x_{M,3}^{1,2}$	$x_{M,1}^{1,3}$	$x_{M,2}^{1,3}$	$x_{M,3}^{1,3}$

(a) Tech data set.

Metabolite	Subject 1			Subject 2			Subject k			Subject 131		
	Replicate 1	Replicate 2	Replicate 3	Replicate 1	Replicate 2	Replicate 3	Replicate 1	Replicate 2	Replicate 3	Replicate 1	Replicate 2	Replicate 3
1	$x_{1,1}^{1}$	$x_{1,2}^{1}$	$x_{1,3}^{1}$	$x_{1,1}^{2}$	$x_{1,2}^{2}$	$x_{1,3}^{2}$	$x_{1,1}^{k}$	$x_{1,2}^{k}$	$x_{1,3}^{k}$	$x_{1,1}^{131}$	$x_{1,2}^{131}$	$x_{1,3}^{131}$
.. $\vdots$	$\vdots$	$\vdots$	$\vdots$	$\vdots$	$\vdots$	$\vdots$	$\vdots$	$\vdots$	$\vdots$	$\vdots$	$\vdots$	$\vdots$
M	$x_{M,1}^{1}$	$x_{M,2}^{1}$	$x_{M,3}^{1}$	$x_{M,1}^{2}$	$x_{M,2}^{2}$	$x_{M,3}^{2}$	$x_{M,1}^{k}$	$x_{M,2}^{k}$	$x_{M,3}^{k}$	$x_{M,1}^{131}$	$x_{M,2}^{131}$	$x_{M,3}^{131}$

(b) BioTech data set.

Metabolite	Subject 1	Subject 2	Subject k	Subject 1130
1	$x_{1,1}$	$x_{1,2}$	$x_{1,k}$	$x_{1,1130}$
.. $\vdots$	$\vdots$	$\vdots$	$\vdots$	$\vdots$
M	$x_{M,1}$	$x_{M,2}$	$x_{M,k}$	$x_{M,1130}$

(c) Bio data set.

Table S2: Data Structure of 3 data sets. Subtables show the hierarchical structure of the Tech, BioTech and Bio data sets. (a) The Tech data set is a triple-layered MS-Metabolomics experiment with the top layer as Operator, middle layer as Spike-in controls and bottom layer as technical replicates. (b) The BioTech data set is a double-layered MS-Metabolomics experiment with the top layer as biological subjects and bottom layer as technical replicates. (c) The Bio data set is a single-layered MS-Metabolomics experiment with only biological subjects.

3 Quartile measures of the parameters

	μ_R	μ_{IR}	σ_R	σ_{IR}	ρ_R	π_1
Quartile 1	3.89	3.19	0.16	0.04	0.992	0.90
Quartile 3	4.13	3.21	0.17	0.05	0.994	0.95

Table S3: First and third quartiles of the parameters. In summary, i) the quartiles of the proportion of reproducible signals (π_1) ranged between 0.90 and 0.95 for 393 sample pairs and 2860 metabolites. ii) The quartiles of the means of the reproducible abundance measures (μ_R), for replicate experiments ranged between 3.89 and 4.13. iii) The quartiles of the means of the irreproducible abundance measures (μ_{IR}), for replicate experiments ranged between 3.19 and 3.21. iv) The quartiles of the standard deviations of the reproducible abundance measures (σ_R) for replicate experiments ranged between 0.16 and 0.17. v) The quartiles of the standard deviations of the irreproducible abundance measures (σ_{IR}) for replicate experiments ranged between 0.04 and 0.05. vi) The quartiles of the Pearson's correlation coefficient of the reproducible abundance measures (ρ_R) between replicate experiments ranged between 0.992 and 0.994.

4 Filtering metabolites by MaRR and RSD

RSD with filtering cutoff 25% (BioTech data set)	Reproducible metabolites identified using 75% cutoff (MaRR procedure)
Technical	69.23
Biological	71.08

Table S4: Filtering metabolites by MaRR with threshold value (75%) of both technical and biological replicates for the BioTech data set. A typical RSD filtering cutoff is 25%. For the BioTech data set, this cutoff does not remove any of the irreproducible metabolites. The RSD values are way the below cutoff percent (25%).

5 MaRR plot

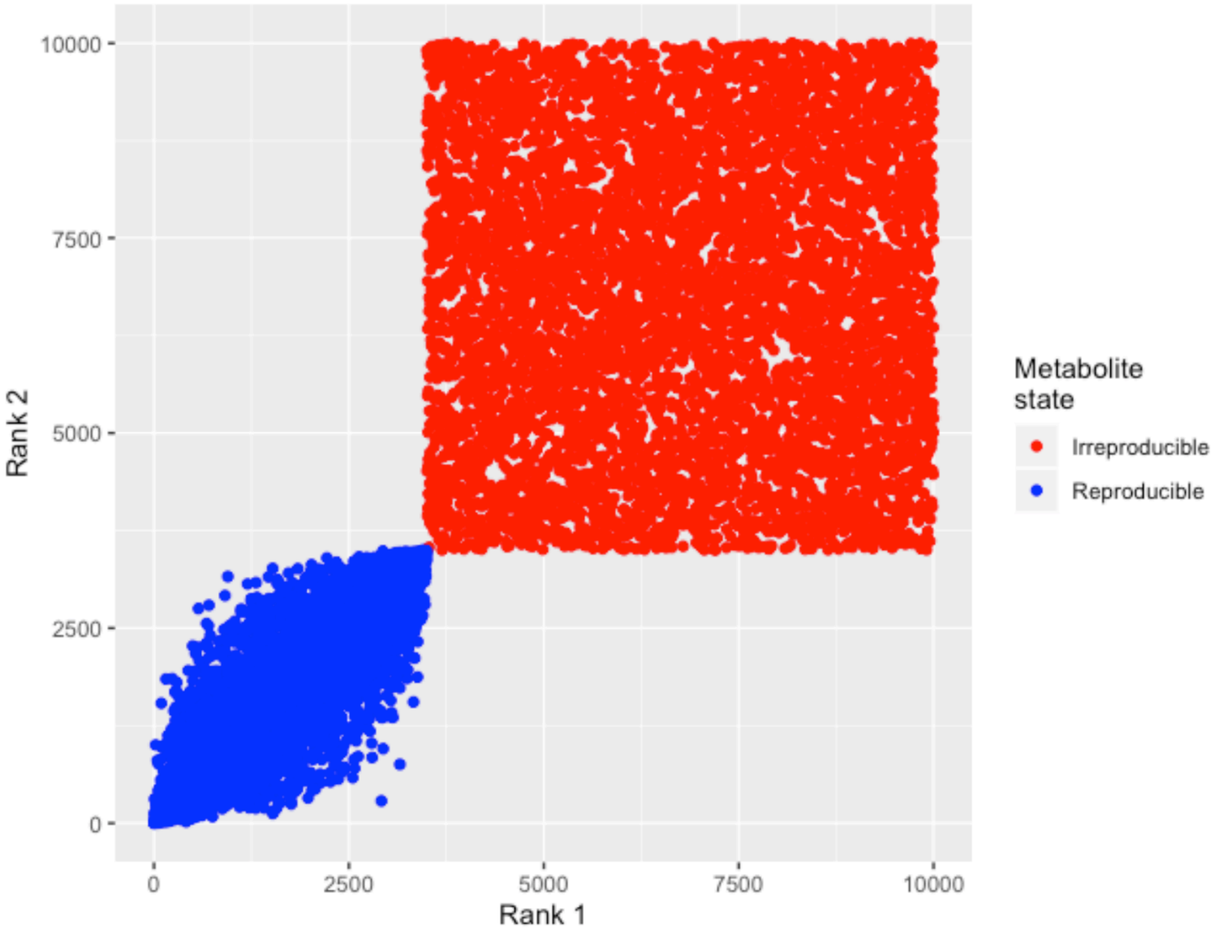

Figure S1: Scatter plot showing rank pairs for 10,000 metabolites. 3,500 of them are reproducible (blue points), while red points indicate irreproducible metabolites (6,500 metabolites). Rank pairs and maximum rank statistics for a sample data set generated under the ideal assumptions with $\pi_1 = 0.35$.

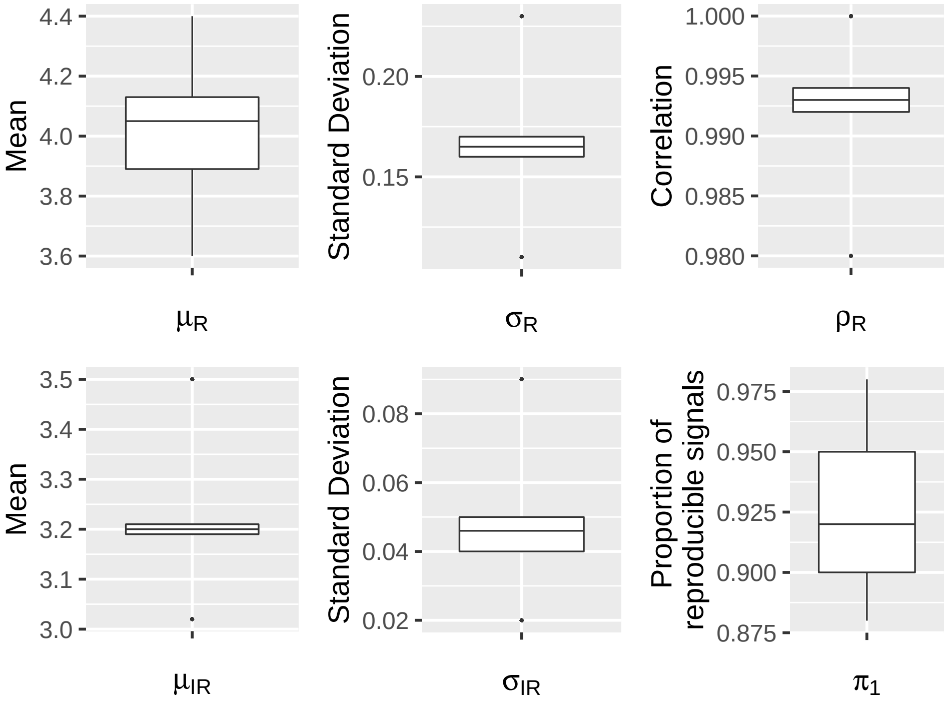

Figure S2: Boxplots showing the spread of the means, standard deviations, correlation and proportion of reproducible/irreproducible signals based on BioTech data set with 393 replicates.

6 Simulation figures

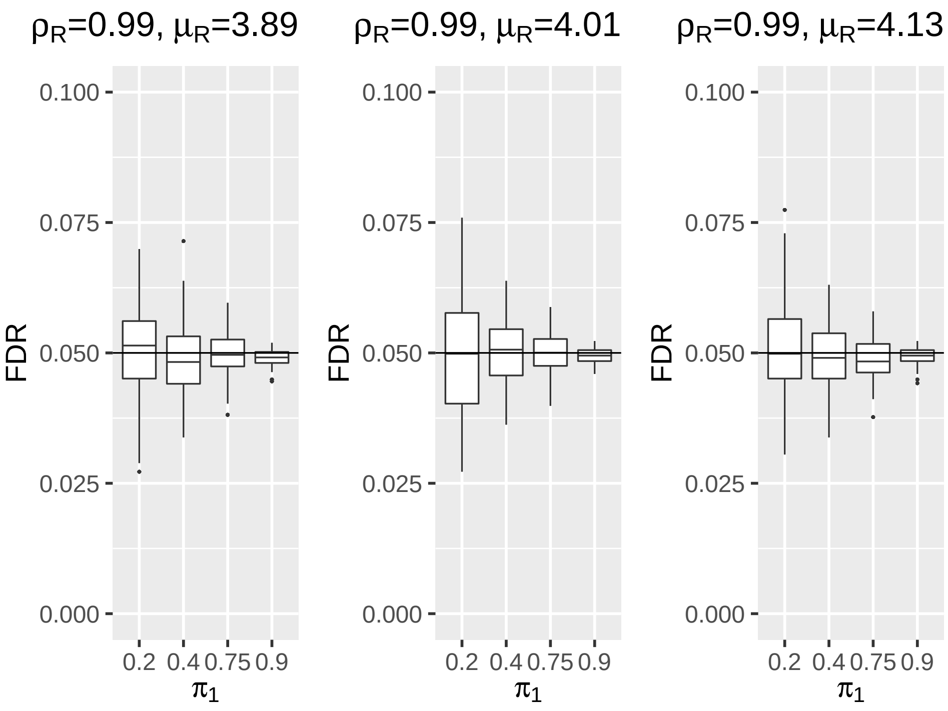

Figure S3: FDRs for simulations I ($\rho_R = 0.99$) based on 1000 simulated datasets in each setting. The horizontal line indicates the target FDR level ($\alpha = 0.05$) for all simulations. Labels along the x-axis describe values of π_1.

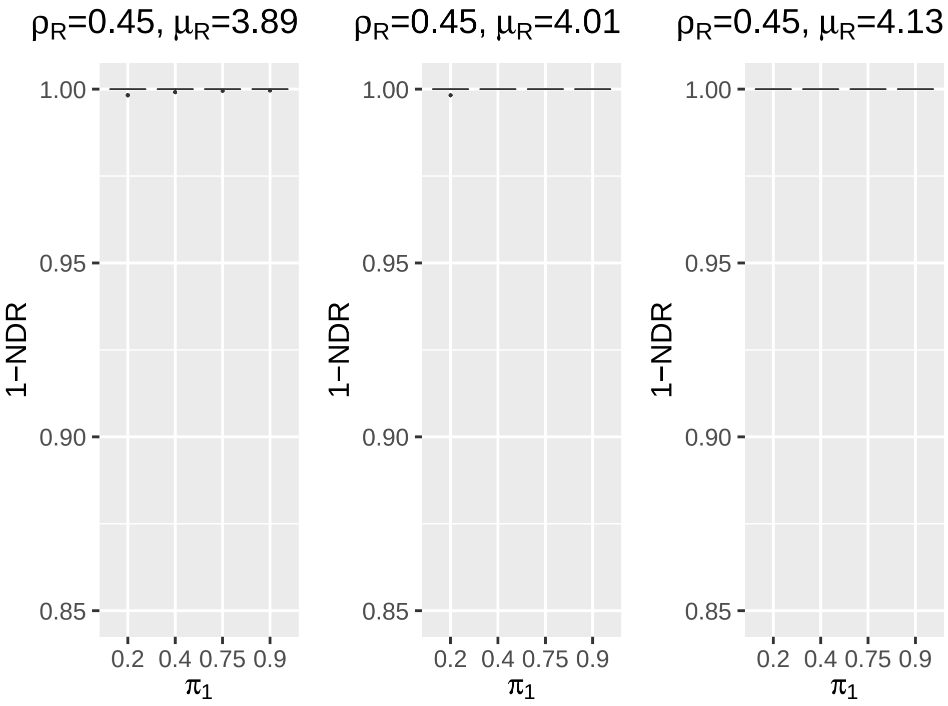

Figure S4: $1 - NDR$ for simulation I ($\rho_R = 0.45$) based on 1000 simulated datasets in each setting. Labels along the x-axis describe values of π_1.

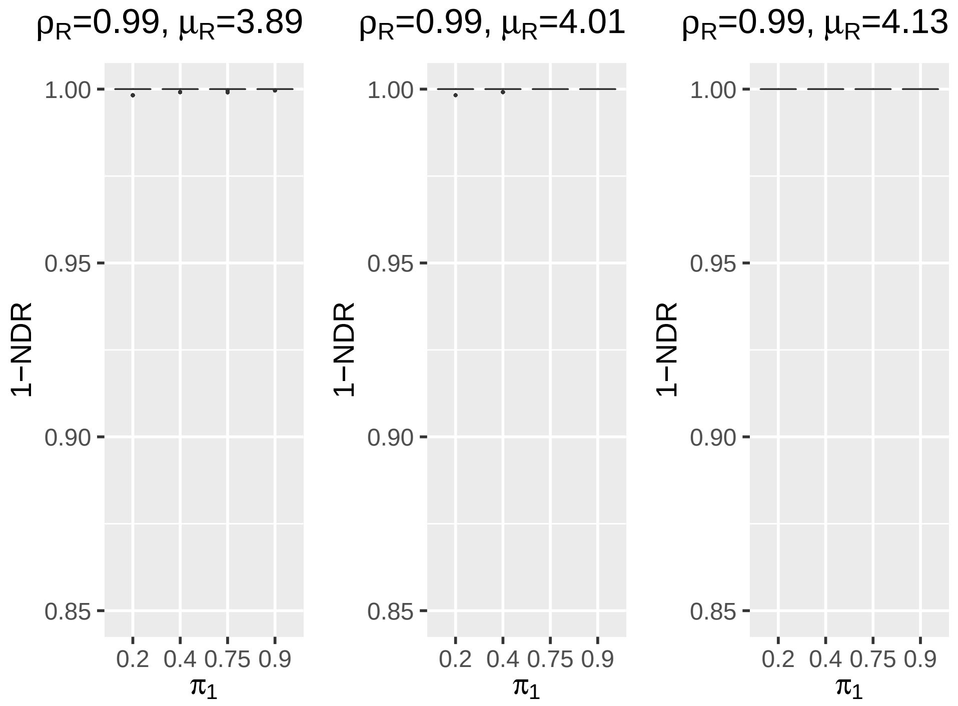

Figure S5: $1 - NDR$ for simulation I ($\rho_R = 0.99$) based on 1000 simulated datasets in each setting. Labels along the x-axis describe values of π_1.

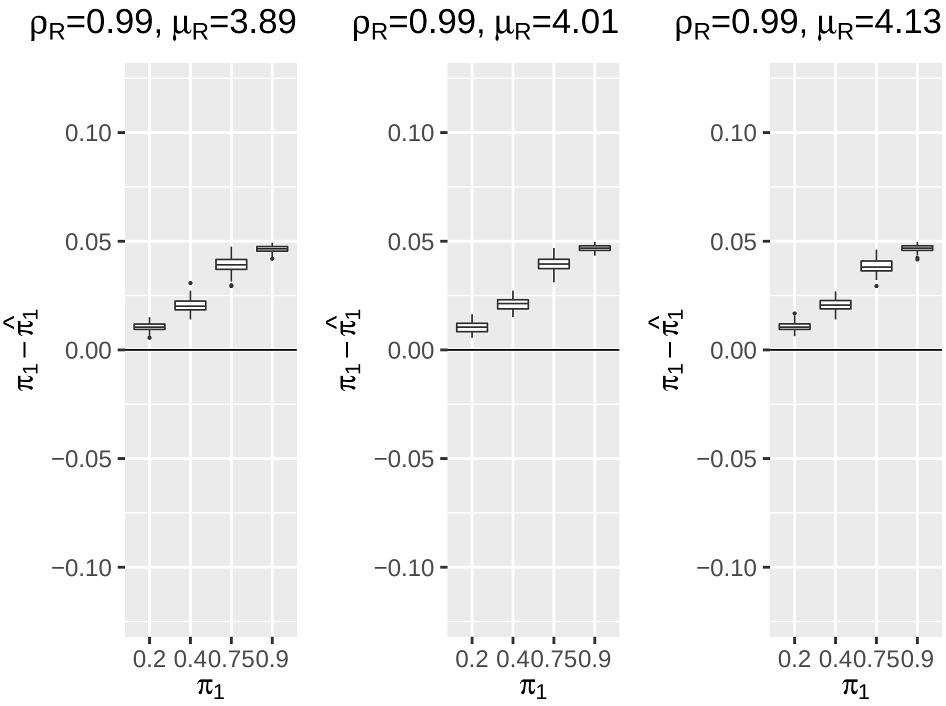

Figure S6: Bias of proportion of reproducible metabolites for simulation I ($\rho_R = 0.99$) based on 1000 simulated datasets in each setting. The horizontal line indicates the target bias (= 0) for all simulations. Labels along the x-axis describe values of π_1.

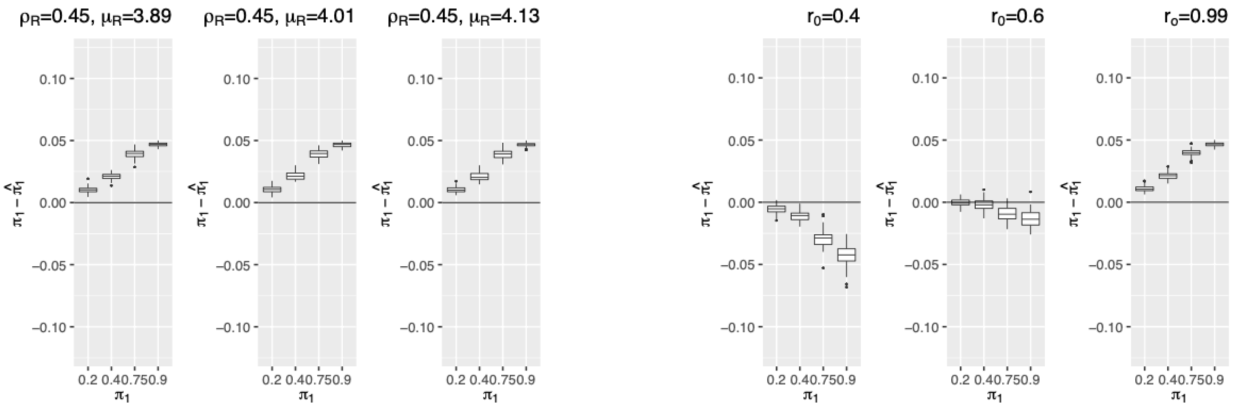

(a) Simulation I ($\rho_R = 0.45$).

(b) Simulation II.

Figure S7: Bias of proportion of reproducible metabolites for simulations I and II based on 1000 simulated datasets in each setting. The horizontal line indicates the target bias (= 0) for all simulations. Labels along the x-axis describe values of π_1.

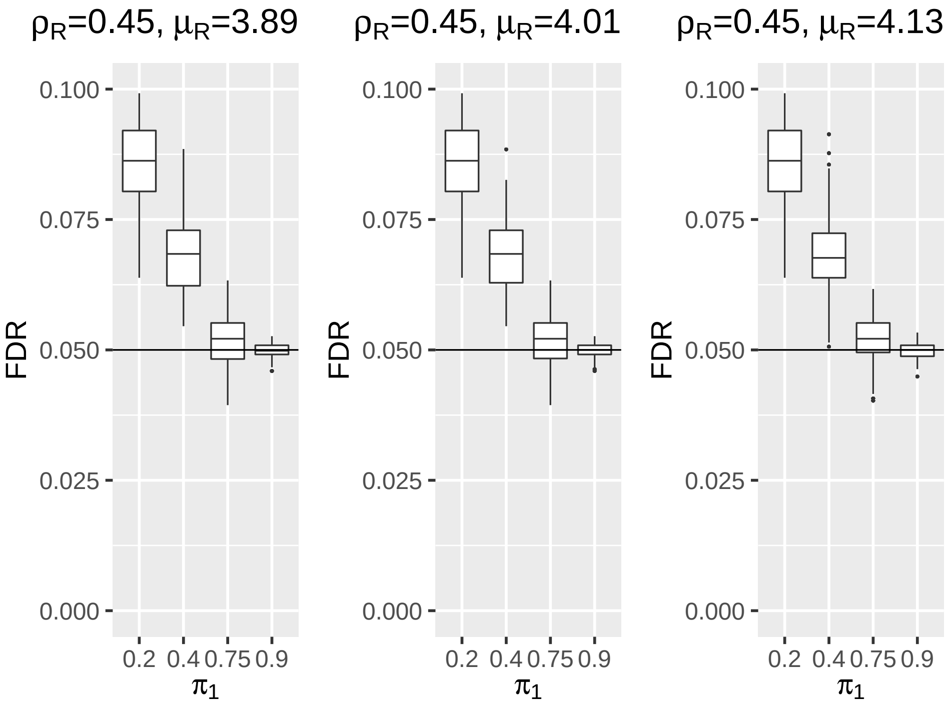

Figure S8: FDRs for simulations III ($\rho_R = 0.45$) based on 1000 simulated datasets in each setting. The horizontal line indicates the target FDR level ($\alpha = 0.05$) for all simulations. Labels along the x-axis describe values of π_1.

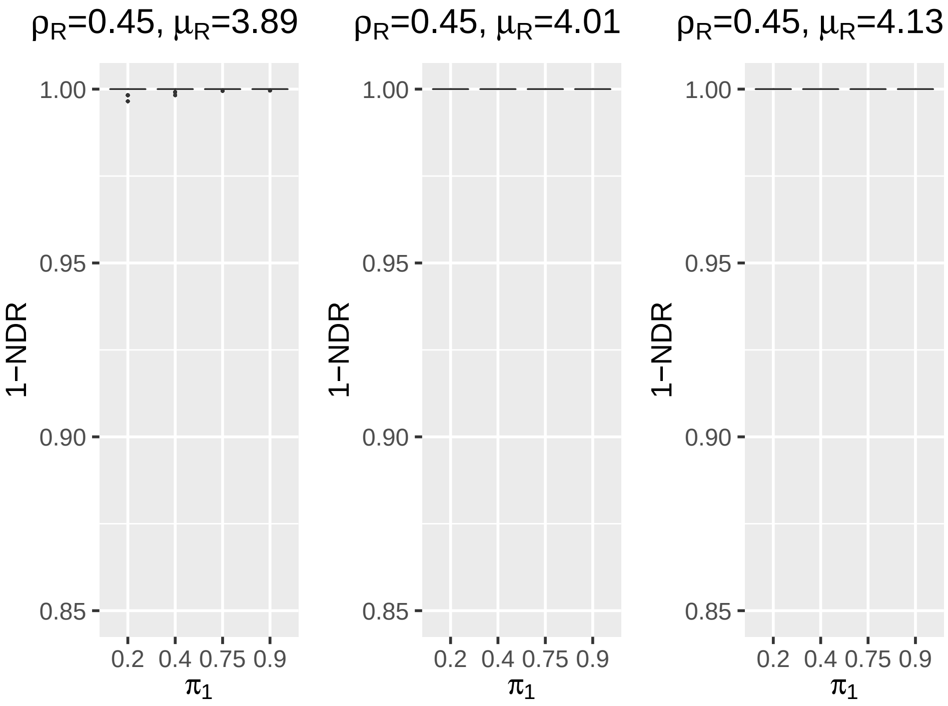

Figure S9: $1 - NDR$ for simulation III ($\rho_R = 0.45$) based on 1000 simulated datasets in each setting. Labels along the x-axis describe values of π_1.

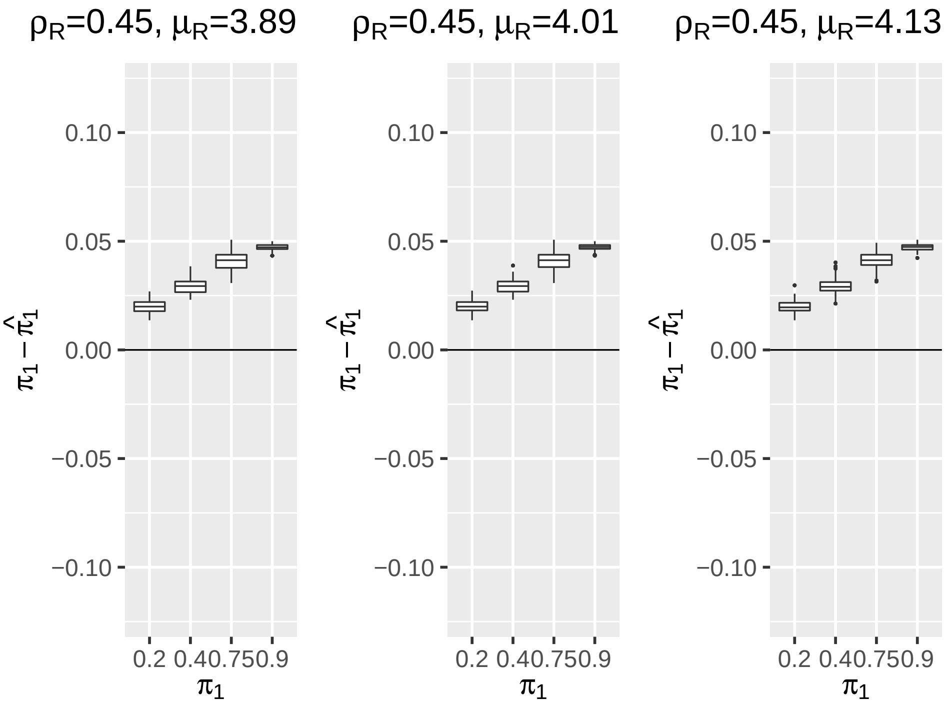

Figure S10: Bias of proportion of reproducible metabolites for simulation III ($\rho_R = 0.45$) based on 1000 simulated datasets in each setting. The horizontal line indicates the target bias (= 0) for all simulations. Labels along the x-axis describe values of π_1.

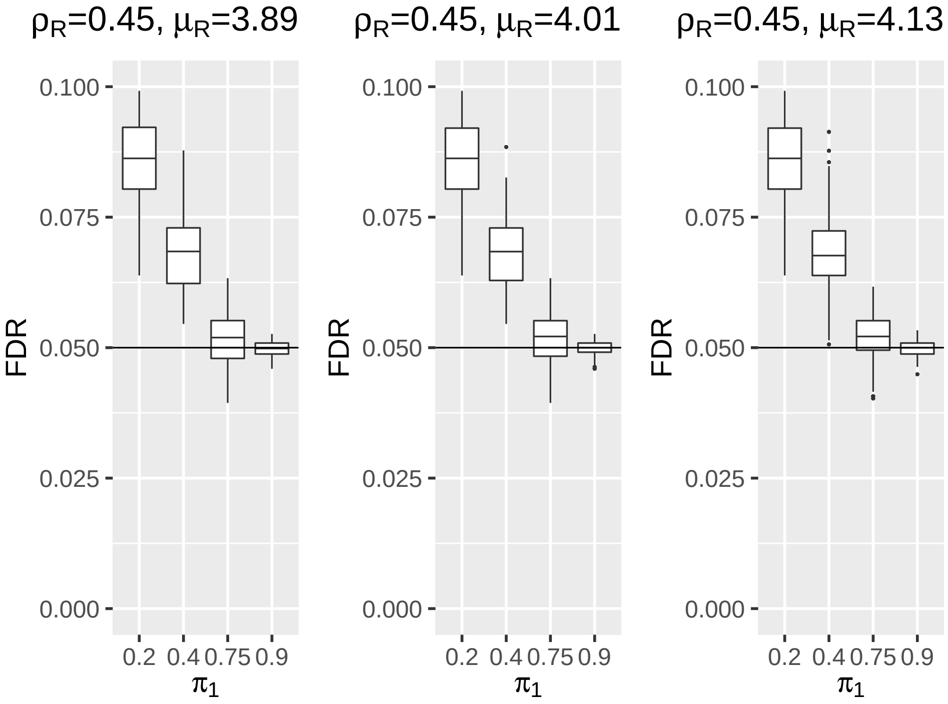

Figure S11: FDRs for simulations III ($\rho_R = 0.99$) based on 1000 simulated datasets in each setting. The horizontal line indicates the target FDR level ($\alpha = 0.05$) for all simulations. Labels along the x-axis describe values of π_1.

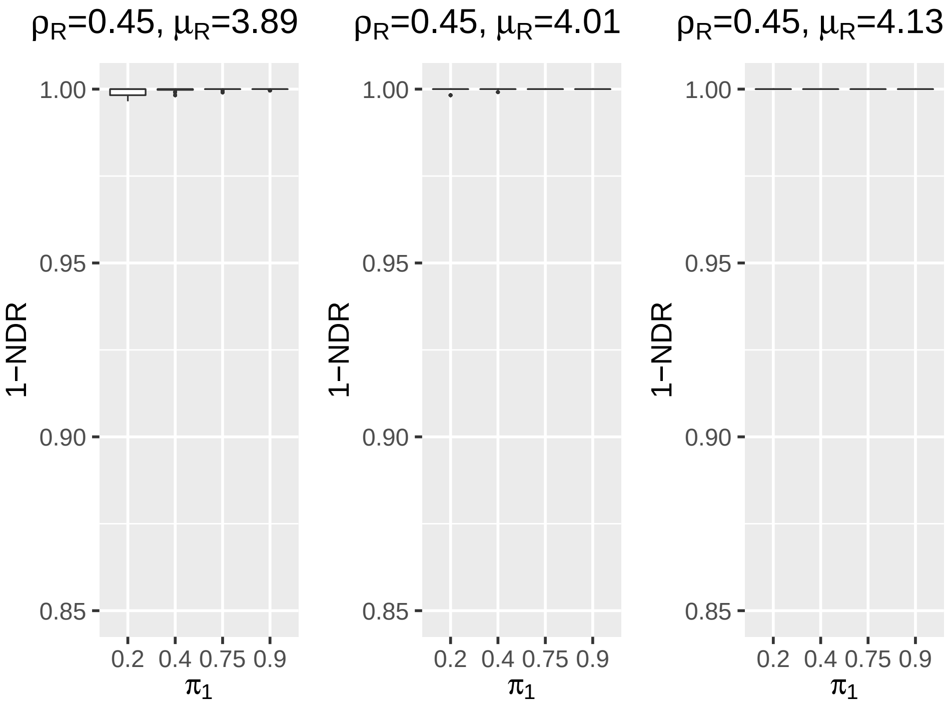

Figure S12: $1 - NDR$ for simulation III ($\rho_R = 0.99$) based on 1000 simulated datasets in each setting. Labels along the x-axis describe values of π_1.

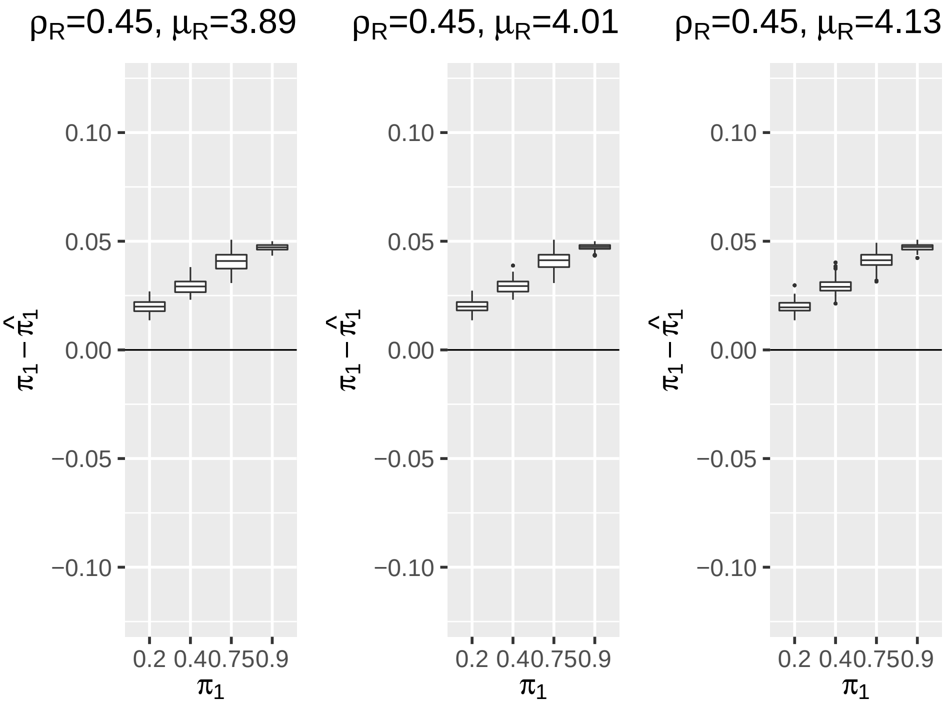

Figure S13: Bias of proportion of reproducible metabolites for simulation III ($\rho_R = 0.99$) based on 1000 simulated datasets in each setting. The horizontal line indicates the target bias (= 0) for all simulations. Labels along the x-axis describe values of π_1.

6.1 Performance evaluation between MaRR and RSD

To compare the performance of the MaRR procedure and RSD, we use a realistic simulation setting from Simulation III with $\mu_R = 4.13$, $\rho_R = 0.99$, and $\pi_1 = 0.90$. We simulate 1000 data sets of metabolite size $M = 2860$. We create a set of thresholds for level of significance α $\{0.01,\ 0.05,\ 0.10,\ 0.20,\ 0.30,\ 0.40,\ 0.50,\ 0.60,\ 0.70,\ 0.80,\ 0.90\}$ to interpret metabolite m to be a reproducible (irreproducible) signal using the MaRR procedure (Equation 10 in the main manuscript). For a direct comparison of the performance, we use the same set of thresholds to identify metabolite m to be a reproducible (irreproducible) signal using RSD. For example, if $\frac{RSD}{100}$ is less than a particular threshold value (say, 0.1), we call metabolite m to be reproducible. In this manner, we compute $Specificity$ ($1 - False\ Positive\ Rate$) and $Sensitivity$ ($True\ Positive\ Rate$) based on the set of threshold values for the MaRR procedure and RSD. For a randomly selected simulated data set (out of 1000 simulated data sets), $1 - Specificity$ and $Sensitivity$ are plotted on the x-axis and y-axis, respectively. The plot is referred to as the Receiver Operating Characteristic (ROC) curve. To compare the performance of the MaRR procedure and RSD based on all the 1000 simulated data sets, we plot AUCs (Area Under the ROC Curve) of the MaRR procedure and RSD from each simulated data set. The ROC curves and AUC boxplots (Figures S14 and S15) indicate that the MaRR procedure performs better in identifying reproducible signals compared to RSD.

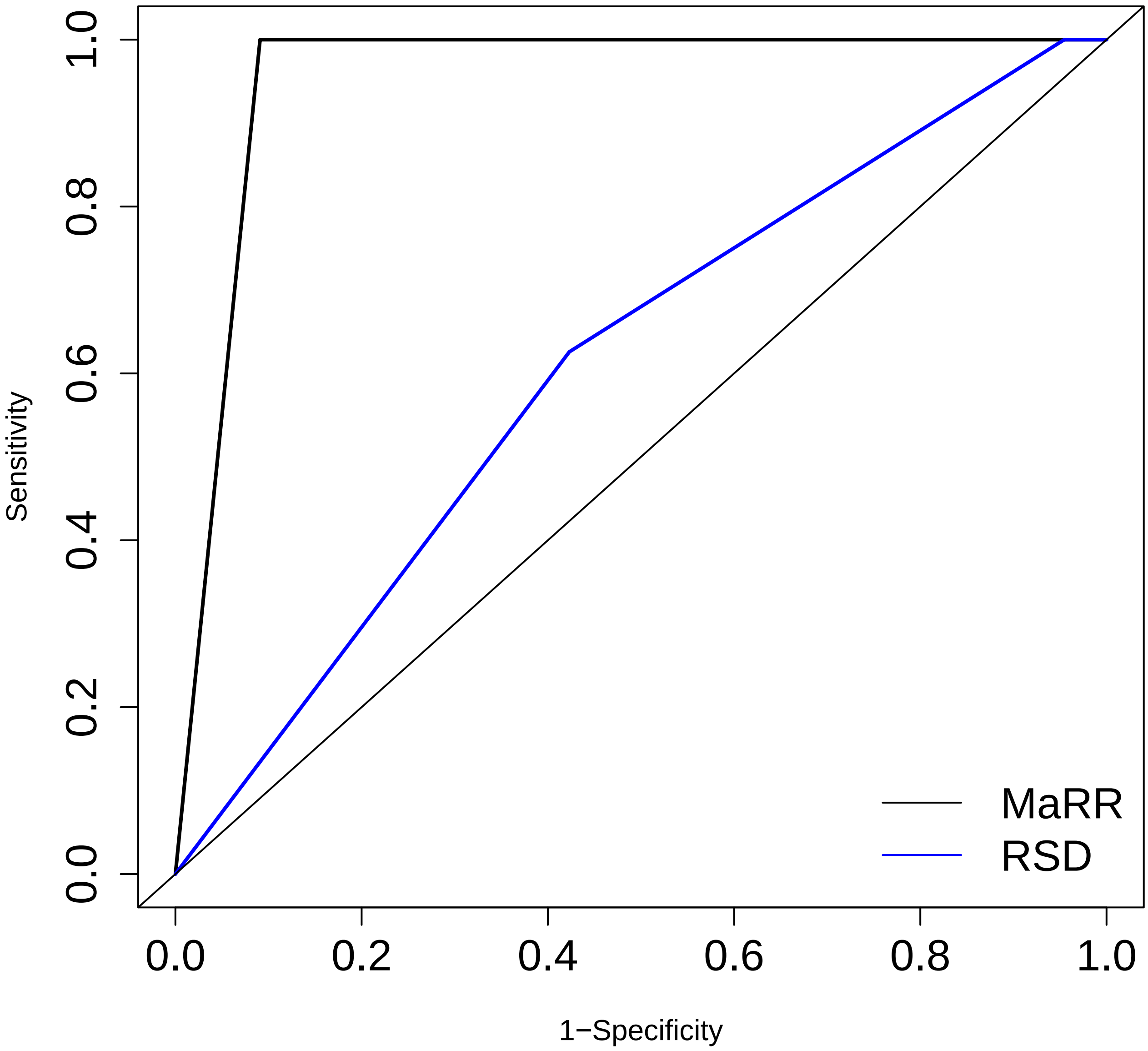

Figure S14: ROC curve of the MaRR procedure and RSD for simulation III with $\mu_R = 4.13$, $\rho_R = 0.99$, and $\pi_1 = 0.90$ based on one randomly selected data set (out of 1000). Each point in the ROC curve correspond to a particular threshold values $\{0.01, 0.05, 0.10, 0.20, 0.30, 0.40, 0.50, 0.60, 0.70, 0.80, 0.90\}$ under which classification (identification of reproducible metabolite) was done.

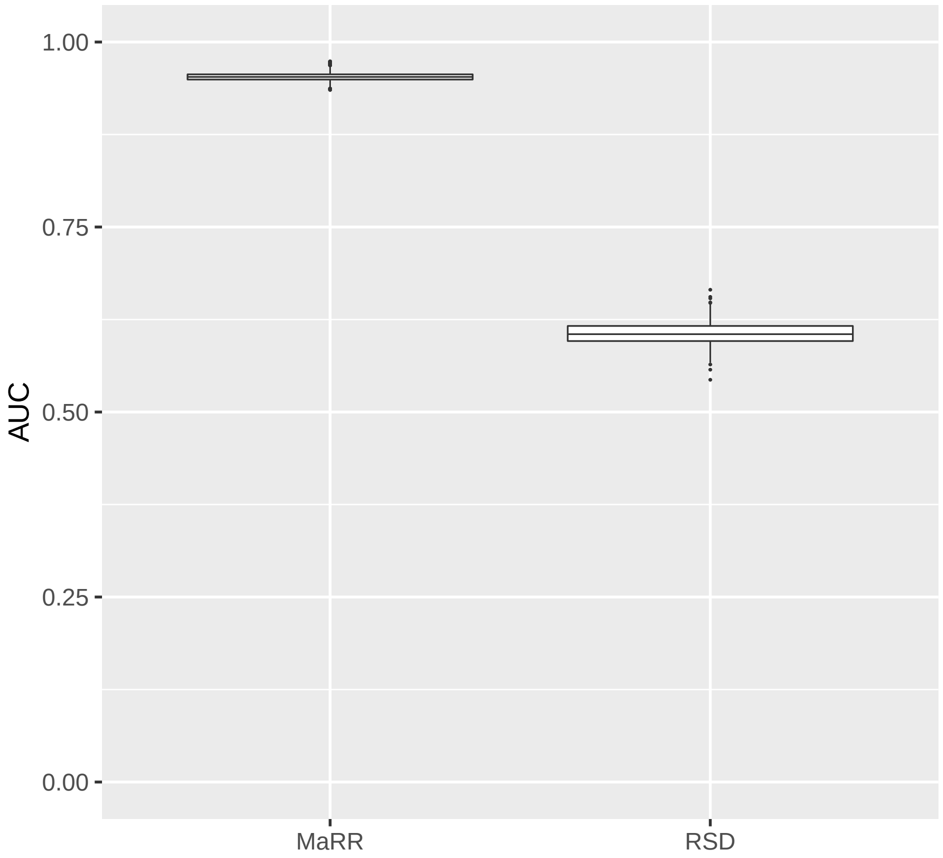

Figure S15: AUC (Area under the ROC) curve of the MaRR procedure and RSD for simulation III with $\mu_R = 4.13$, $\rho_R = 0.99$, and $\pi_1 = 0.90$ based on 1000 simulated data sets. For each simulated data set, each point in the ROC curve correspond to a particular threshold values $\{0.01,\ 0.05,\ 0.10,\ 0.20,\ 0.30,\ 0.40,\ 0.50,\ 0.60,\ 0.70,\ 0.80,\ 0.90\}$ under which reproducible signals are classified.

7 Reproducibility of sample pairs per metabolite

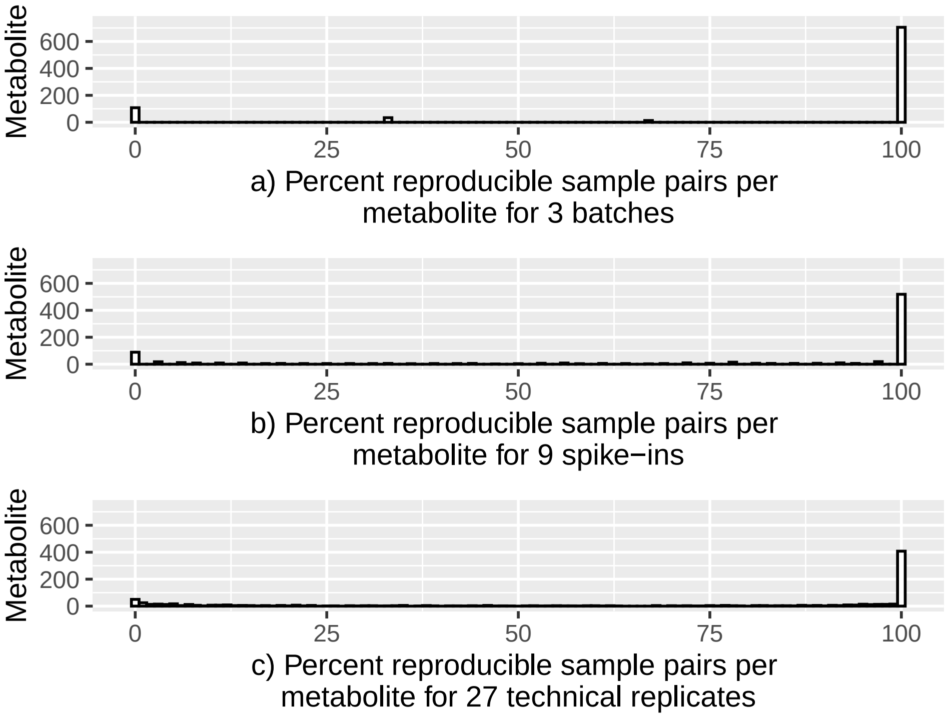

Figure S16: Histograms showing reproducible sample pairs for three layers of the Tech data set. These histograms are in percent scale where the x-axes denote the percent scale of reproducible sample pairs for all metabolites. a) Top layer (batches): three pairs of batches. b) Middle layer (spike-ins): nine pairs of spike-ins. c) Bottom layer (technical replicates): 27 pairs of technical replicates.

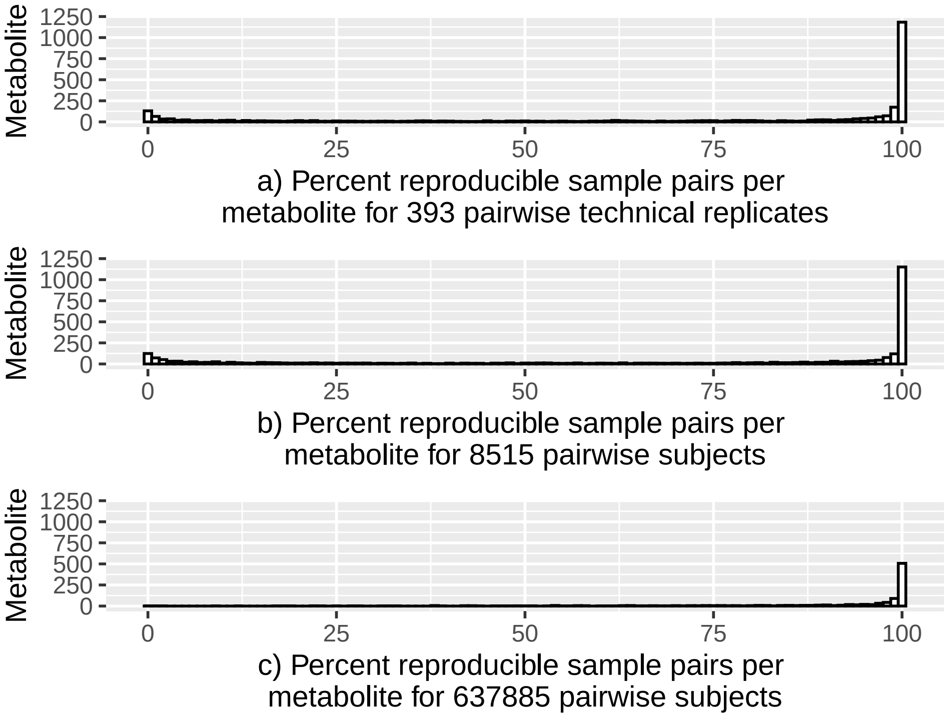

Figure S17: Histograms showing reproducible sample pairs per metabolite for BioTech and Bio data sets. These histograms are in percent scale where the x-axes denote the percent scale of reproducible sample pairs. Reproducible sample pairs a) 393 technical replicate sample pairs (BioTech), b) 8515 biological replicate sample pairs (BioTech), and c) 637885 biological replicate sample pairs (Bio).

8 Using MaRR to compare data processing methods

8.1 Imputation- BPCA and Normalization- median

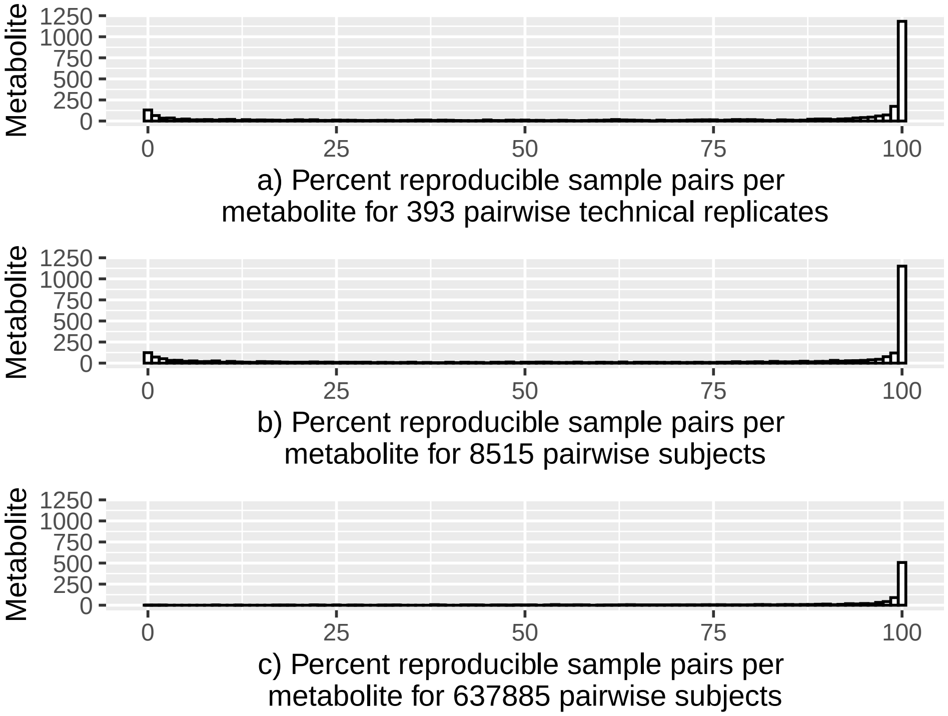

Figure S18: Histograms showing reproducible sample pairs per metabolite for BioTech and Bio data sets. These histograms are in percent scale where the x-axes denote the percent scale of reproducible sample pairs. Reproducible sample pairs a) 393 technical replicate sample pairs (BioTech), b) 8515 biological replicate sample pairs (BioTech), and c) 637885 biological replicate sample pairs (Bio).

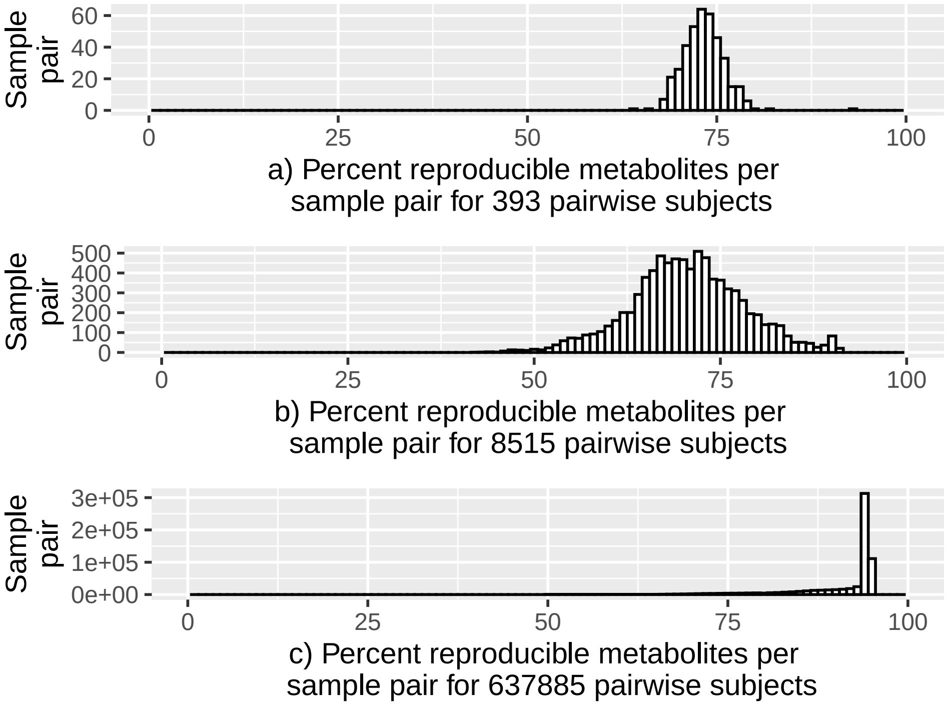

Figure S19: Histograms showing reproducible metabolites per sample pair for BioTech and Bio data sets. These histograms are in percent scale where the x-axes denote the percent scale of reproducible metabolites. Reproducible metabolites a) 393 technical replicate sample pairs (BioTech), b) 8515 biological replicate sample pairs (BioTech), and c) 637885 biological replicate sample pairs (Bio).

8.2 Imputation- kNN and Normalization- median

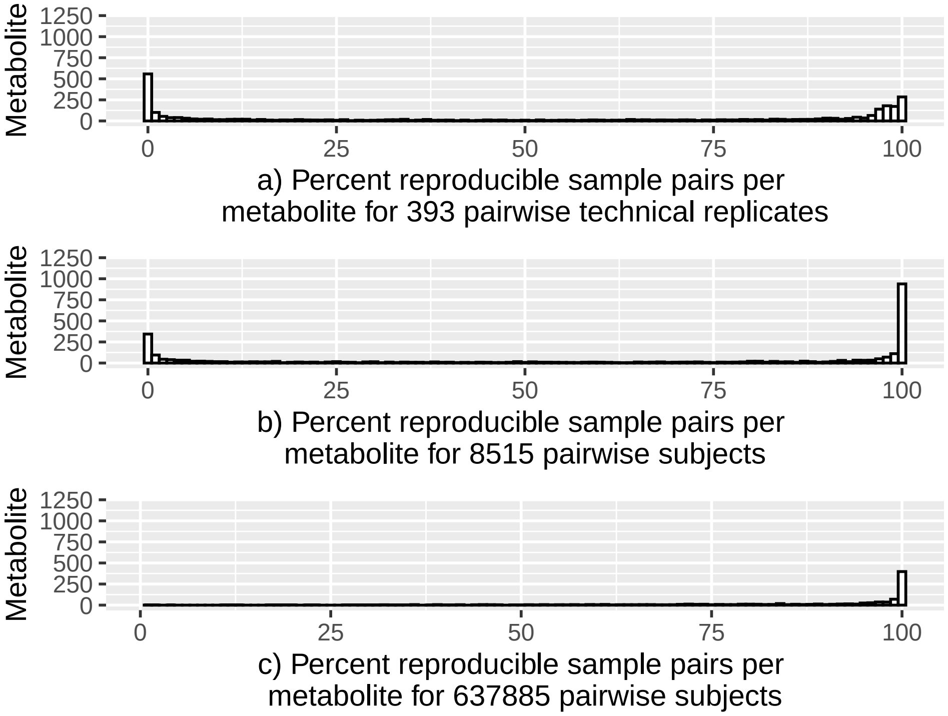

Figure S20: Histograms showing reproducible sample pairs per metabolite for BioTech and Bio data sets. These histograms are in percent scale where the x-axes denote the percent scale of reproducible sample pairs. Reproducible sample pairs a) 393 technical replicate sample pairs (BioTech), b) 8515 biological replicate sample pairs (BioTech), and c) 637885 biological replicate sample pairs (Bio).

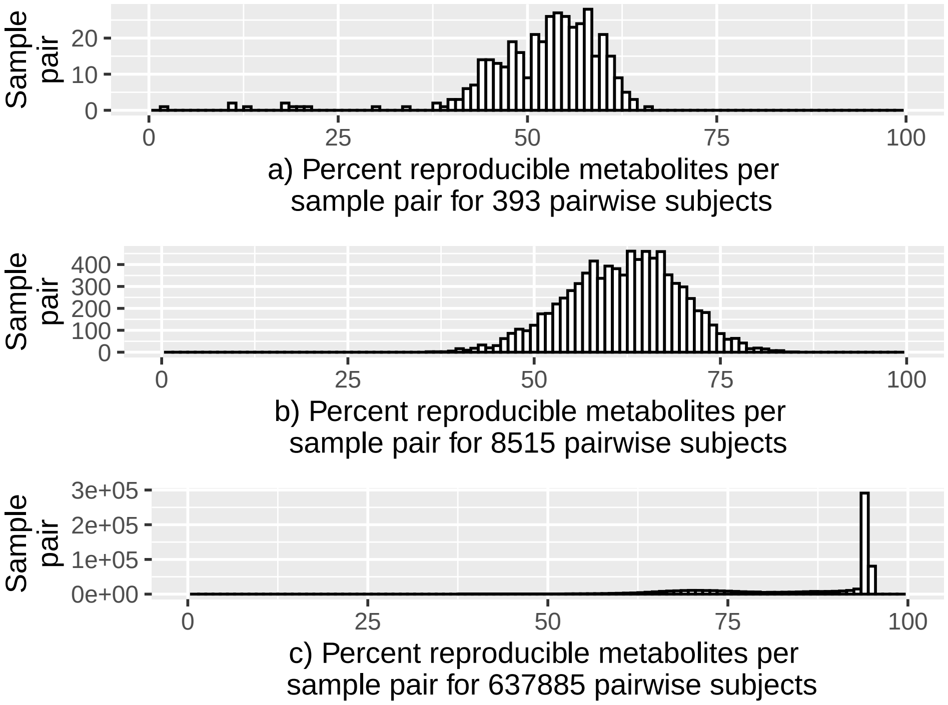

Figure S21: Histograms showing reproducible metabolites per sample pair for BioTech and Bio data sets. These histograms are in percent scale where the x-axes denote the percent scale of reproducible metabolites. Reproducible metabolites a) 393 technical replicate sample pairs (BioTech), b) 8515 biological replicate sample pairs (BioTech), and c) 637885 biological replicate sample pairs (Bio).

8.3 Imputation- kNN and Normalization- quantile

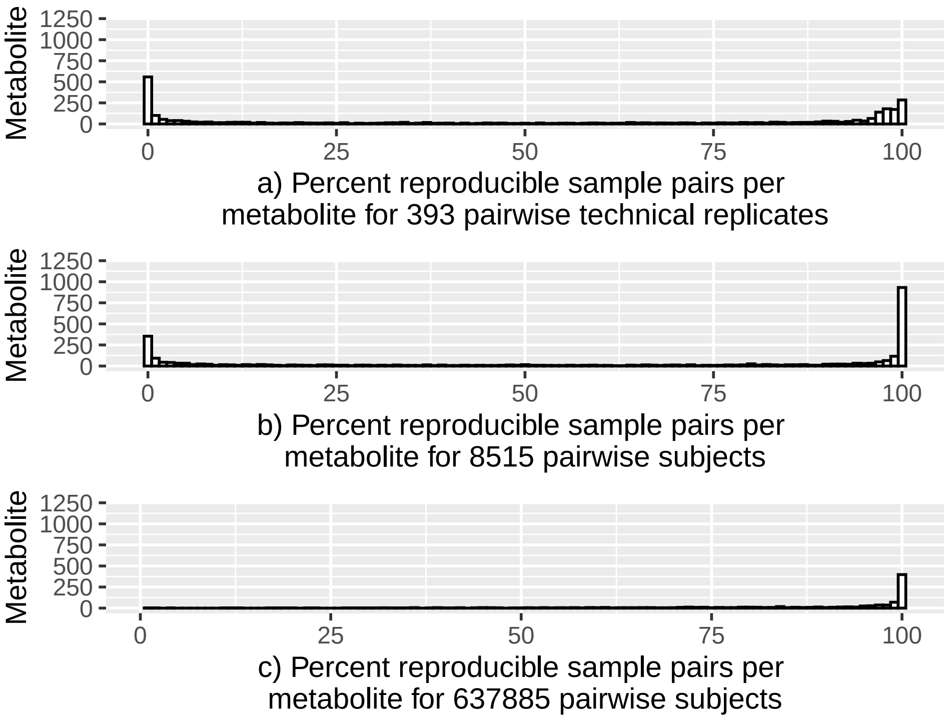

Figure S22: Histograms showing reproducible sample pairs per metabolite for BioTech and Bio data sets. These histograms are in percent scale where the x-axes denote the percent scale of reproducible sample pairs. Reproducible sample pairs a) 393 technical replicate sample pairs (BioTech), b) 8515 biological replicate sample pairs (BioTech), and c) 637885 biological replicate sample pairs (Bio).

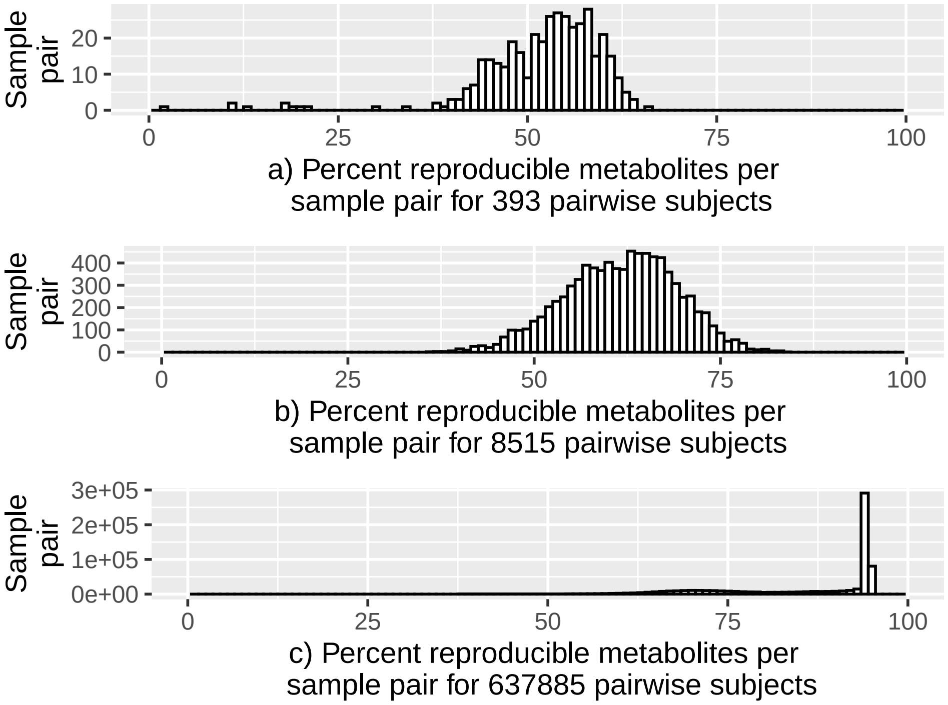

Figure S23: Histograms showing reproducible metabolites per sample pair for BioTech and Bio data sets. These histograms are in percent scale where the x-axes denote the percent scale of reproducible metabolites. Reproducible metabolites a) 393 technical replicate sample pairs (BioTech), b) 8515 biological replicate sample pairs (BioTech), and c) 637885 biological replicate sample pairs (Bio).

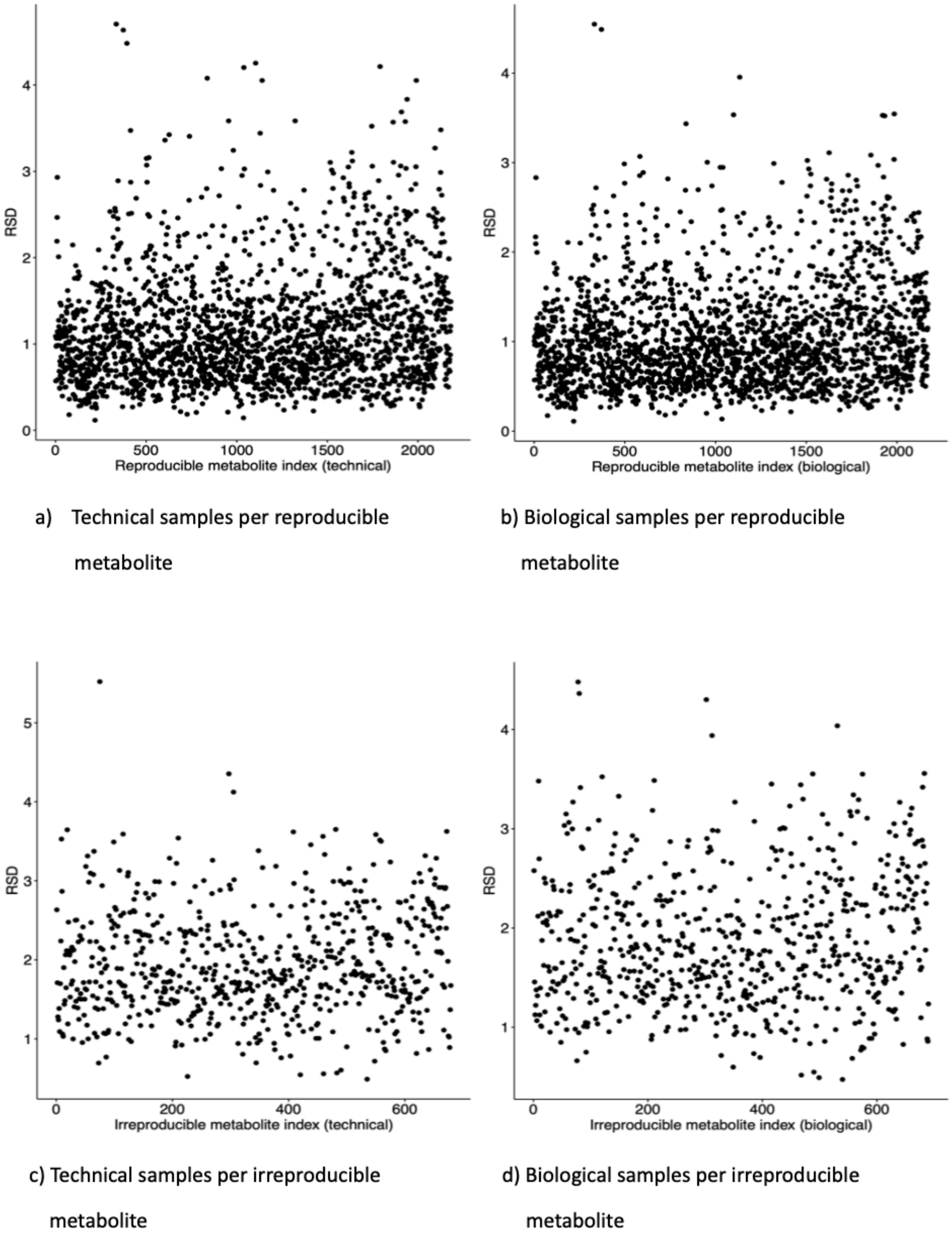

a) Technical samples per reproducible metabolite

b) Biological samples per reproducible metabolite

c) Technical samples per irreproducible metabolite

d) Biological samples per irreproducible metabolite

Figure S24: Scatterplots showing RSD of samples per metabolite index for the BioTech data set. Metabolites are filtered by MaRR with threshold value (> 75% (<= 75%) implies reproducible (irreproducible) metabolites). The horizontal (x) axes denote the metabolite indices and the vertical (y) axes denote the RSD values per metabolite.

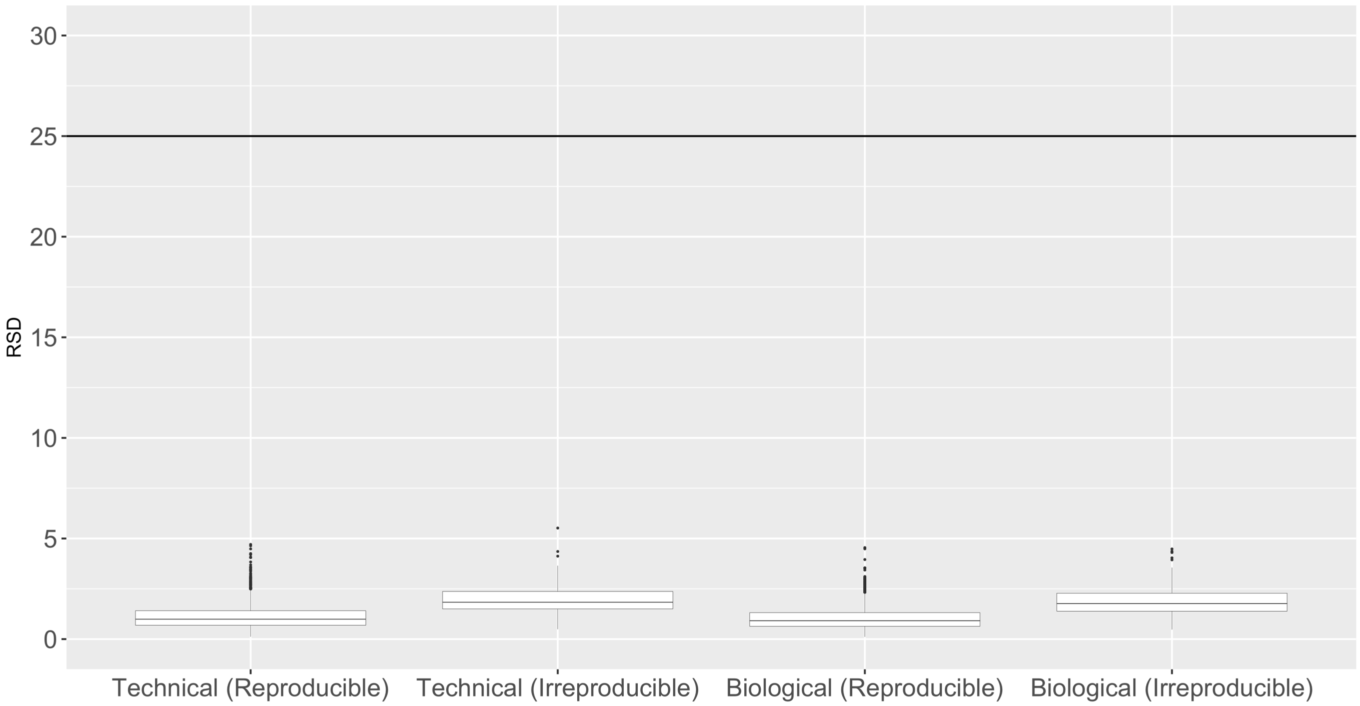

Figure S25: Boxplots showing the reproducible and irreproducible metabolites of both technical and biological replicates filtered by RSD for the BioTech data set. Metabolites are filtered by MaRR with threshold value (> 75% (<= 75%) implies reproducible (irreproducible) metabolites). A typical RSD filtering cutoff is 25% (horizontal black line). For the BioTech data set, this cutoff does not remove any of the irreproducible metabolites. The RSD values are way the below cutoff percent (25%).

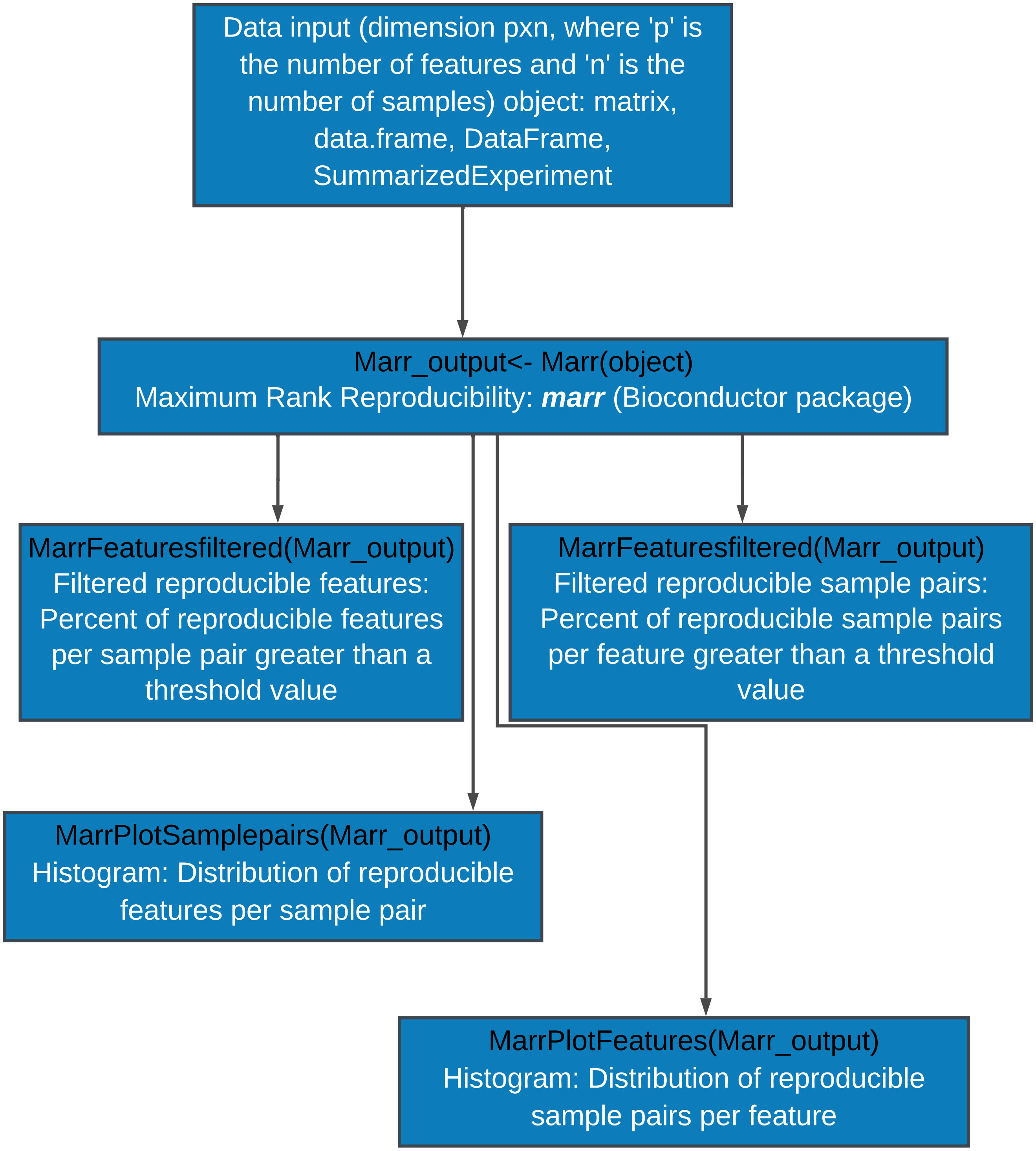

Figure S26: A simple flowchart of the *marr* Bioconductor package. The methods described in the manuscript are implemented in the open-source R package *marr*, which is freely available from Bioconductor at `http://bioconductor.org/packages/marr`. The *marr* package includes comprehensive help files for each function, as well as a package vignette demonstrating a complete example study design.

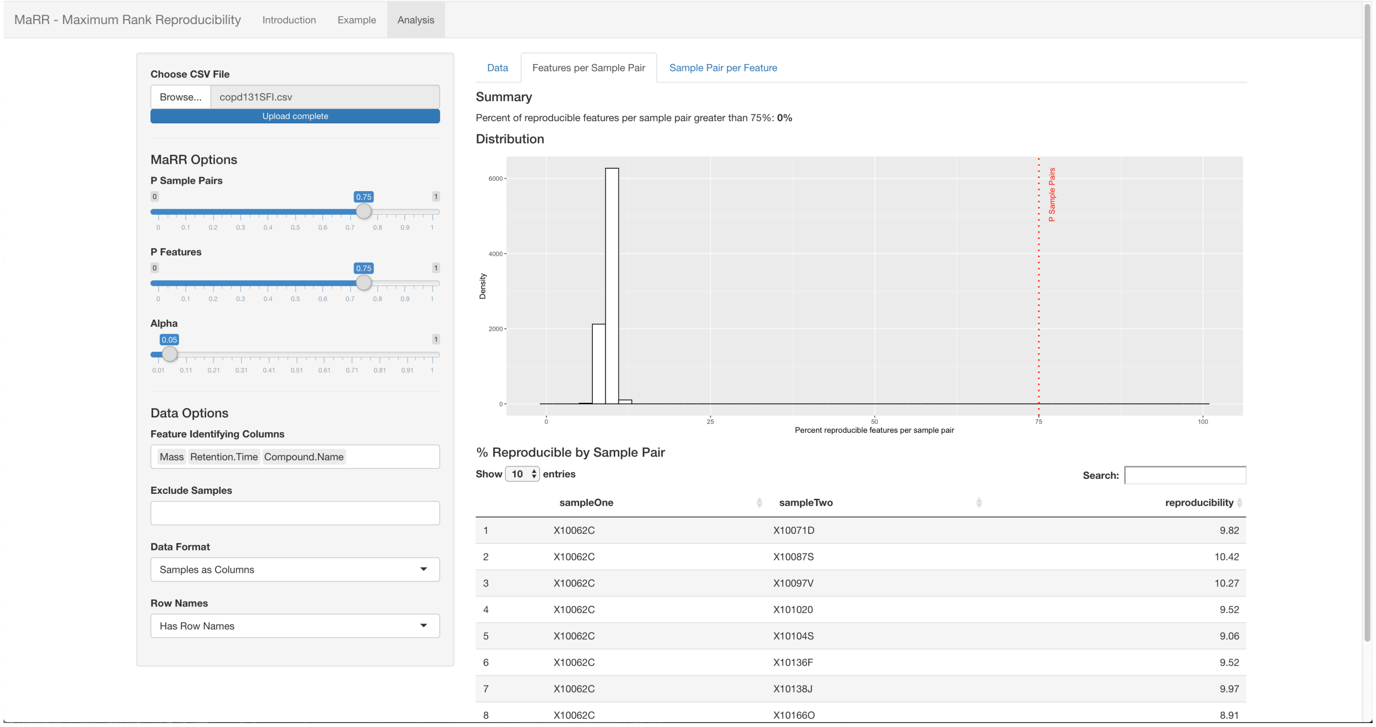

Figure S27: Illustrative snapshot of the marr Shiny-based Web application, called marr Shiny , for dynamic interaction with MS-metabolomics data that can run on any Web browser and requires no prior programming knowledge [`https://maxmcgrath.shinyapps.io/marr/`]. Percent reproducible features per sample pair.

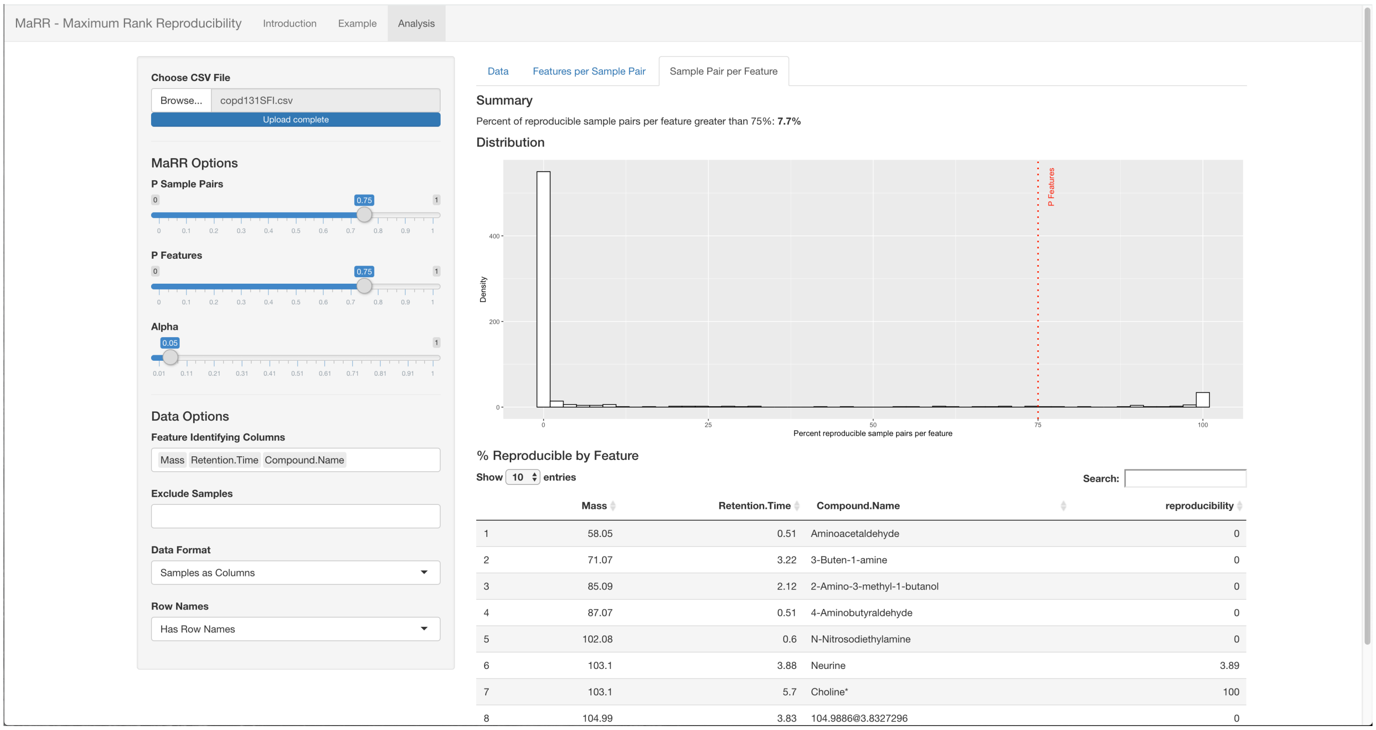

Figure S28: Illustrative snapshot of the marr Shiny-based Web application, called marr Shiny , for dynamic interaction with MS-metabolomics data that can run on any Web browser and requires no prior programming knowledge [https://maxmcgrath.shinyapps.io/marr/]. Percent reproducible sample pairs per feature.
